# Polyunsaturated fatty acids promote M2-like TAM deposition via dampening RhoA-YAP1 signaling in the ovarian cancer microenvironment

**DOI:** 10.1186/s40164-024-00558-8

**Published:** 2024-08-28

**Authors:** Huogang Wang, Mingo MH Yung, Yang Xuan, Fushun Chen, Waisun Chan, Michelle KY Siu, Runying Long, Shuo Jia, Yonghao Liang, Dakang Xu, Zhangfa Song, Stephen KW Tsui, Hextan YS Ngan, Karen KL Chan, David W Chan

**Affiliations:** 1 Department of Obstetrics & Gynaecology, School of Clinical Medicine, LKS Faculty of Medicine, The University of Hong Kong, Hong Kong, P.R. China; 2 Department of Colorectal Surgery, Sir Run Run Shaw Hospital, School of Medicine, Zhejiang University, Hangzhou, 310016 Zhejiang P.R. China; 3 Eye Center, The Second Affiliated Hospital, School of Medicine, Zhejiang University, Hangzhou, 310009 P.R. China; 4 School of Biomedical Sciences, The Chinese University of Hong Kong, Hong Kong, P.R. China; 5 Faculty of Medical Laboratory Science, Ruijin Hospital, School of Medicine, Shanghai Jiao Tong University, Shanghai, 200030 P.R. China; 6 School of Medicine, The Chinese University of Hong Kong, Shenzhen, 518172 Guangdong P.R. China

**Keywords:** Tumor microenvironment, Immunometabolism, TAM polarization, Ovarian cancer and ascites, Polyunsaturated fatty acids, RhoA-YAP1 signaling

## Abstract

**Background:**

Peritoneal metastases frequently occur in epithelial ovarian cancer (EOC), resulting in poor prognosis and survival rates. Tumor-associated-macrophages (TAMs) massively infiltrate into ascites spheroids and are multi-polarized as protumoral M2-like phenotype, orchestrating the immunosuppression and promoting tumor progression. However, the impact of omental conditioned medium/ascites (OCM/AS) on TAM polarization and its function in tumor progression remains elusive.

**Methods:**

The distribution and polarization of TAMs in primary and omental metastatic EOC patients’ tumors and ascites were examined by m-IHC, FACS analysis, and immunofluorescence. QPCR, immunofluorescence, FACS analysis, lipid staining assay, ROS assay, and Seahorse real-time cell metabolic assay characterized TAMs as being polarized in the ascites microenvironment. The oncogenic role of TAMs in tumor cells was demonstrated by co-cultured migration/invasion, proliferation, and spheroid formation assays. Mechanistic studies of the regulations of TAM polarization were performed by using RNA-Seq, GTPase pull-down, G-LISA activation assays, and other biochemical assays. A Yap1 macrophages (MФs) conditional knockout (cKO) mouse model demonstrated the roles of YAP1 in TAM polarization status and its pro-metastatic function. Finally, the anti-metastatic potential of targeting TAMs through restoring YAP1 by pharmacological agonist XMU MP1 was demonstrated in vitro and in vivo.

**Results:**

Abundant polyunsaturated fatty acids (PUFAs) in OCM/AS suppressed RhoA-GTPase activities, which, in turn, downregulated nuclear YAP1 in MФs, leading to increased protumoral TAM polarization accompanied by elevated OXPHOS metabolism. Abolishment of YAP1 in MФs further confirmed that a higher M2/M1 ratio of TAM polarization could alleviate CD8^+^ T cell infiltration and cytotoxicity in vivo. Consistently, the loss of YAP1 has been observed in EOC metastatic tissues, suggesting its clinical relevance. On the contrary, restoration of YAP1 expression by pharmaceutical inhibition of MST1/2 induced conversion of M2-to-M1-like polarized MФs, elevating the infiltration of CD8^+^ T cells and attenuating tumor growth.

**Conclusion:**

This study revealed that PUFAs-enriched OCM/AS of EOC promotes M2-like TAM polarization through RhoA-YAP1 inhibition, where YAP1 downregulation is required for accelerating protumoral M2-like TAM polarization, thereby causing immunosuppression and enhancing tumor progression. Conversion of M2-to-M1-like polarized MФs through Yap1 activation inhibits tumor progression and contributes to developing potential TAMs-targeted immunotherapies in combating EOC peritoneal metastases.

**Supplementary Information:**

The online version contains supplementary material available at 10.1186/s40164-024-00558-8.

## Background

Tangible evidence has suggested that metastasis is responsible for cancer morbidity and mortality, whose progression is driven not only by the endogenous force of tumor cells but also by the exogenous shaping of the peripheral microenvironment [1]. The tumor microenvironment (TME) is a complicated ecosystem that is comprised of cancer cells and various supporting cellular and non-cellular components, acting as the crucial regulator of tumor development and metastatic progression [2]. The tumor-infiltrating immune cells (TIICs) are the major cellular components in the composition of TME, which can exercise antitumor activity but are sometimes deprived by TME [3, 4]. TME choreographers the distribution and polarization of TIICs, causing a uniquely immunosuppressed microenvironment, which in turn promotes tumor growth and metastasis [4, 5]. Hence, understanding the role of TME in educating tumor-associated immune cells enables us to explore novel strategies for efficient anticancer treatment.

Tumor-associated macrophages (TAMs) are the most abundant population of TIICs in TME [6, 7]. TAMs exhibit a high degree of functional plasticity and can rapidly adapt to TME through the polarization of MФs into either protumoral (M2-like) or tumoricidal (M1-like) activity [7, 8]. M2-like TAMs display trophic functions promoting metastasis and mediating immunosuppressive TME to debilitate antitumor immune responses [9]. In contrast, M1-like TAMs can activate the adaptative immune response and kill cancer cells [7, 10]. Emerging evidence has suggested that TME modulates TAM polarization to lean toward protumoral activity, associated with poor prognosis in various tumors [11]. However, the underlying mechanism of lipids-enriched TME in modulating TAM polarization remains obscure.

Clinical evidence has revealed that epithelial ovarian cancer (EOC) possesses precedential metastatic tropism for the adipose-enriched omentum and accumulation in ascites, suggesting that the lipid-enriched TME is pivotal in facilitating tumor progression and metastasis [1]. Recent studies indicate lipid-enriched TME fuels tumor cells and exacerbates EOC progression through lipid metabolic reprogramming [12, 13]. Simultaneously, lipid-enriched TME affects the differentiation of immune cells and antitumor activity while maintaining a suppressive immune microenvironment, which facilitates tumor progression [14]. The ascites microenvironment is frequently hijacked by immune cell infiltrate (ICI) that exhibits immunosuppression and debilitates antitumor immune responses [15]. Limited glucose availability but abundance of lipids induce a metabolic shift in immune cells, which impairs their tumoricidal functions [14]. This metabolic adaptation is consequently linked to cytotoxic CD8^+^ T cell dysfunction across many human cancer types and mouse models [14].

Cell functions and statutes are directly influenced by nutrient conditions via modulating multiple signaling pathways, such as Hippo signaling [16, 17]. The Hippo signaling cascade is integral in orchestrating cell proliferation, differentiation, and survival, thereby maintaining organogenesis and homeostasis. Dysfunctional Hippo pathway signaling is implicated in the pathogenesis of various diseases, notably oncogenesis and immunological dysregulation. Both Yes-associated protein (YAP) and transcriptional coactivator with PDZ-binding motif (TAZ) serve as key transcriptional effectors of the Hippo signaling cascade, frequently causing unchecked activation in tumor cells [18]. Although the functions of Hippo signaling constituents in regulating malignant transformation within tumor cells are well-documented, their roles within immune cells remain poorly understood.

Here, we report that the abundant polyunsaturated fatty acids (PUFAs) in the OCM/AS play a crucial role in promoting protumoral M2-like TAM polarization, which, in turn, creates an immunosuppressive TME in promoting ovarian cancer peritoneal metastases. Using OCM/AS derived from EOC patients and mouse models with conditional Yap1 knockout (cKO) in murine MΦs, we demonstrated that the dominant PUFAs promoted protumoral M2-like TAM polarization by inactivating RhoA-YAP1 signaling cascade and *Yap1*^−/−^ TAMs enhanced EOC metastatic properties. Notably, we provided valid evidence that restoring nuclear Yap1 by targeting MST1/2 in TAMs inhibited peritoneal metastases accompanied by increased M1-like polarized MФs and elevated cytotoxic CD8^+^ T cell infiltration. Taken together, these findings manifest that the abundant PUFAs are crucial for facilitating protumoral M2-like TAM polarization in lipid-enriched TME and promoting EOC tumor growth and progression.

## Methods

### Cell culture

Human high-grade ovarian serous adenocarcinoma (HGSOC) cell lines OVKATE and OVSAHO cells were purchased from the Japanese Collection of Research Bioresources Cell Bank (JCRB Cell Bank, Tokyo, Japan). OVKATE and OVSAHO cells, known for their high metastatic potential, were used to study the migration and invasion capacities in vitro. Human clear cell subtype ovarian cancer cell line ES-2 cells was purchased from the American Type Culture Collection (ATCC, Manassas, Virginia, USA). Human ovarian cancer adenocarcinoma cell line A2780cp cells was obtained from Prof. Benjamin Tsang, University of Ottawa, Canada. Different ovarian cancer cell lines, such as ES2 and A2780cp cells, were used for tumorigenic assays, such as spheroid formation. A murine high-grade serous subtype ovarian cancer cell line, ID8 cells, was obtained from Dr. Katherine F. Roby, The University of Kansas, USA. As detailed in our previous study, the murine ovarian cancer cell line ID8 cells were utilized to establish an in vivo peritoneal metastases model in cKO Yap1 mice [19]. Human leukemia monocytic cell line THP-1 cells and human pro-monocytic cell line U937 cells were purchased from Cell Line Service (CLS, Eppelheim, Germany). All cell lines were incubated at 37 °C in a humidified incubator containing 5% CO_2_. ES-2 and ID8 cells were cultured in Dulbecco’s modified Eagle medium (DMEM). A2780cp, OVKATE, and OVSAHO cells were cultured in RPMI 1640 Medium (Gibco). All full media were supplemented with 10% fetal bovine serum (FBS) (Gibco) and 1% penicillin-streptomycin (P/S) (Invitrogen).

### Macrophage stimulation

In various experiments, two human monocyte cell lines, THP-1 and U937 cells, were used as established in vitro models for human macrophages. Primary human monocytes isolated from buffy coats and subsequently validated were used to corroborate the presence of these phenotypes. Likewise, primary murine monocytes and conditionally knocked-out (CKO) murine macrophages were utilized to confirm the results in vivo. The induction protocol is described below. Murine bone marrow-derived macrophages (BMDMs) were obtained as previously described [20] and cultured in complete DMEM/F12 (Thermo Fisher Scientific) containing 10 ng/mL murine M-CSF (LPEPROTECH). Murine peritoneal macrophages (PEMs) were harvested from mice treated with an *i.p*. injection of 3% Brewer thioglycollate medium (Thermo Fisher Scientific), as previously reported [21]. Human PBMC-derived monocytes were positively selected by CD14^+^ magnetic beads (Miltenyi Biotec) from the buffy coat (Blood Storage & Issue Section Hong Kong Red Cross) and were cultured in ImmunoCult^TM^-SF Macrophage Medium (STEMCELL) with 50 ng/mL M-CSF for six days to polarize resting macrophages (M0 MΦs). THP-1 cells were cultured in RPMI with 200 ng/mL phorbol 12-myristate 13-acetate (PMA) (Abcam), and U937 cells were cultured with 200 ng/mL PMA for 24 h and then in the medium without PMA to polarize M0 MΦs. Human M1 MΦs were differentiated in 20 pg/mL LPS (Enzo Life Sciences) plus 20 ng/mL IFN-γ (PEPROTECH), and M2 MΦs were differentiated in 20 ng/mL IL13 (PEPROTECH) and 20 ng/mL IL4 (PEPROTECH). Murine M1 MΦs were activated with murine 100 ng/mL LPS (Sigma Aldrich) plus 10 ng/mL IFN-γ (PEPROTECH); M2 MΦs were activated with murine 10 ng/mL IL4 (PEPROTECH) plus 10 ng/mL IL13 (PEPROTECH). M0 MΦs treated with either omental conditioned medium (OCM) or ascites (AS) were defined as M1-like TAMs or M2-like TAMs respectively based on their polarization markers and functions.

### Human specimens

OCM was prepared as previously described [12]. Omentum was freshly collected, rinsed with PBS, and then minced into small pieces. The omental mixture was subsequently added to the RPMI medium and incubated for 24 h. The omental tissues were removed by centrifugation and filtration. To selectively remove lipids, Cleanascite™ Lipid Removal Reagent (Biotech Support Group) was added to the OCM according to the manufacturer’s suggestions. The ascites samples from EOC patients were filtered and stored at 4 °C for further studies.

### Animal studies

Yap1^fl/fl^ mice (C57BL/6J, Yap1tm1.1Dupa/J, Stock No. 027929) were purchased from The Jackson Laboratory, and LysM-Cre mice (C57BL/6J, B6.129P2-Lyz2tm1(cre)Ifo/J-Stock No: 004781) were a gift from Dr. RCC CHANG (The University of Hong Kong, China). The LysM-Cre mice were crossed with Yap1^fl/fl^ mice to produce heterozygotes. The F4-F6 generations of heterozygotes were interbred to generate homozygous Yap1 cKO (*Yap1*^−/−^) myeloid cell lineage, including wild-type (WT) mice, according to our previous report [19]. All mice were maintained under specific pathogen-free (SPF) conditions in the Minimal Disease Area (MDA), the Centre for Comparative Medicine Research (CCMR), LKS Faculty of Medicine, The University of Hong Kong. The entire animal study was performed according to guidelines approved by The Committee on the Use of Live Animals in Teaching and Research of The University of Hong Kong (CULATR number: 5489-20).

### RNA-Seq analysis

As previously described, THP-1 and U937 monocytes were induced to differentiate into M0 MФs using PMA [22]. Subsequently, M0 MФs were polarized into M2 MФs with IL-13/IL-4 or into M1 MФs with LPS/IFN-γ. These differentiated cells were then treated with either OCM or a control medium. Following treatment, the cells were harvested for RNA extraction and sequencing. RNA integrity and concentration were assessed using NanoDrop One/OneC, Invitrogen Qubit, and Agilent 4200 TapeStation systems before RNA-seq library preparation. Transcriptomic profiling of macrophages was conducted by HaploX Biotechnology Co., Ltd. (Shenzhen, China). For constructing the transcriptome sequencing library, each sample yielded 10–20 gigabases of 200 bp paired-end reads using the Illumina PE150 platform. Quality control of sequencing reads was performed using FASTP version 0.20.0. Alignment to the human reference genome (GRCh38) was accomplished with HISAT2 version 2.1.0, utilizing a GTF file to annotate splice junctions. On average, 96% of reads are mapped to the reference genome, with a unique alignment rate of 91%. Gene expression quantification from the raw sequencing data was performed using HISAT2. Subsequently, gene expression levels were normalized in terms of fragments per kilobase of transcript per million mapped reads (FPKM) using EdgeR and DESeq2. Differential expression analysis and fold change estimation between experimental conditions were conducted to employ the trimmed mean of M-values (TMM) normalization method. The RNA-seq dataset is deposited in the NCBI Gene Expression Omnibus under accession number GSE175552.

### Transfection of siRNA

THP-1 MΦs were seeded onto 6-well plates and incubated for 24 h. Cells were then transfected with YAP1 siRNA and control siRNA (or RhoA siRNA and control siRNA) using HiPerFect Transfection Reagent (QIAGEN) according to the manufacturer’s suggestions. Two days after transfection, the transfected cells were collected for Western blot analysis to assess the knockdown efficacy of YAP1 and RhoA. This was accomplished using YAP1 Human siRNA Oligo Duplex (Locus ID 10413, OriGene) and RHOA Human siRNA Oligo Duplex (Locus ID 387, OriGene) to specifically target YAP1 and RhoA, respectively.

### RNA extraction and real-time PCR

Total RNA was isolated from the cell pellet utilizing TRIzol reagent, and reverse transcription was conducted using SuperScript™ VILO™ Master Mix (Thermo Fisher Scientific, CO), according to our prior publication [19]. Quantitative real-time PCR (qPCR) was executed employing TaqMan probes from the TaqMan Gene Expression Assays (Applied Biosystems) following the manufacturer’s protocol. The qPCR thermocycling conditions included an initial denaturation at 95 °C for 5 min, followed by 40 cycles of 15 s at 95 °C and 1 min at 60 °C for annealing and extension. Relative gene expression was quantified using human 18 S (Assay ID: Hs03003631_g1) and murine GAPDH (Assay ID: Hs99999905_m1) as endogenous controls. The 2-ΔΔCt method was employed to calculate relative expression levels. The sequences of all primers used are provided in Supplementary Table S1.

### Western blot analysis

Protein lysates were prepared in cell lysis buffer (Cell Signaling Technology), and separated via SDS–PAGE before being transferred to FL-PVDF membranes. The blots were incubated overnight at 4 °C with the following primary antibodies: rabbit anti-YAP1 (Elabscience), rabbit anti-Lamin B1 (ProteinTech), rabbit anti-MST1 (Cell Signaling Technology), rabbit anti-RhoA (Cell Signaling Technology), rabbit anti-Cdc42 (Cell Signaling Technology), rabbit anti-RAC1 (Thermo Fisher Scientific), rabbit anti-TAZ (Abcam), mouse anti-beta Actin (Thermo Fisher Scientific) and mouse anti-Tubulin (Sigma Aldrich). Next, the membranes were washed with TBST and subjected to fluorescence-conjugated secondary antibodies (LI-COR IRDye 680/800 CW, 1:15,000 dilution). Fluorescence visualization was performed using an LI-COR Odyssey CLx Imager (Lincoln). All antibodies are listed in Table S1.

### Cytoplasmic and nuclear protein extraction

Macrophages were harvested using trypsin-EDTA and centrifuged at 500x g for 5 min. Subsequently, the cells underwent a single wash with PBS and were centrifuged again at 500x g for 2–3 min. After discarding the supernatant, the resultant cell pellets were processed to extract cytoplasmic and nuclear proteins, following the procedures outlined by the NE-PER Nuclear and Cytoplasmic Extraction Reagents kit (Thermo Fisher Scientific) [19].

### Multiplex IHC staining

An Opal 8-color kit (Akoya Biosciences) was used for m-IHC according to the manufacturer’s protocol. Formalin-fixed, paraffin-embedded (FFPE) sections were dewaxed, rehydrated, and fixed with 10% paraformaldehyde solution (PFA) for 20 min. Then, the slides were rinsed with TBST and AR6 buffer and were microwaved within the AR6 buffer (Akoya Biosciences). The slides were cooled to room temperature (RT), washed with TBST, and blocked with Opal antibody Diluent/blot (Akoya Biosciences) for 10 min. The primary antibody was added and incubated at 4℃ overnight. The slides were washed with TBST. Then, a Polymer HRP (Ms + Rb) secondary antibody was added into slides and incubated at RT for 10 min. Opal dye (1:100) was applied for 10 min after the washes. Then, the slides were washed and treated with the microwave. This process was repeated five more times using various antibodies. All antibodies used are listed in Table S1. Nuclei were stained with DAPI (Akoya Biosciences) and mounted with medium (Abcam). Living Image Software and InForm 2.4.2. were used to analyze the m-IHC data.

### Cytosolic ROS measurement

The cytosolic ROS analyses were performed according to the manufacturer’s protocol. Briefly, cells were incubated in a humidified chamber at 37 °C with 5% CO_2_ for 30 min with DCFDA / H2DCFDA (Abcam, Cat# ab113851) in a cell culture medium. After incubation, the cells were washed and detected by fluorescence spectroscopy with 485 nm/535 nm within 2 h of staining.

### Metabolic phenotyping

According to the manufacturer’s instructions, the metabolic status of macrophages was quantified using the Agilent Seahorse XF Cell Mito Stress Test kit (Catalog #103015-100). A total of 5 × 10^5^ MΦs per well (> 3 wells per sample) were added to poly-D-lysine-coated seahorse 96-well plates and preincubated in Seahorse XF media (unbuffered RPMI + 10 mM L-glutamine + 10 mM sodium pyruvate + 25 mM glucose) (Agilent Technologies) at 37 °C for a minimum of 30 min in the absence of CO_2_. OCR and ECAR were measured under basal conditions and after the addition of the following drugs in Seahorse XF Cell Mito Stress Test Kit (Agilent Technologies): 1 µM oligomycin, 1.5 µM fluorocarbonyl cyanide phenylhydrazone (FCCP), and 0.5 µM rotenone + antimycin A as indicated. Measurements were taken using a 96-well Extracellular Flux Analyzer (Seahorse Bioscience).

### Active GTPase pull-down and G-LISA activation assays

Active Rho Pull-Down and Detection Kit (Thermo Fisher Scientific, Cat# 16116) were adopted according to the manufacturer’s protocol. RhoA was used as the primary antibody in the assay (Cell Signaling Technology, Cat# BK124-S). The activation of RhoA was measured using G-LISA activation assay kits (Cytoskeleton, Cat# BK124-S) according to the manufacturer’s instructions.

### Isolation of TAMs

Tumors or paired omentum metastatic tissues were harvested and cut into small pieces. Tumor lysates were digested in PBS, and omentum lysates were digested in serum-free DMEM containing Liberase DL(Roche), Liberase TL (Roche), and DNaseI (Sigma Aldrich) for 1–18 h at 37 °C. The cells were filtered and stained at 4℃. TAMs were sorted as CD45^+^ (Invitrogen™), CD11b^+^ (Biolegend), CD14^+^ (Biolegend), CD163^+^ (Biolegend), CD3^−^ (Biolegend), CD19^−^ (Biolegend), and CD56^−^ (Biolegend) with a BD FACSAria SORP and analyzed for CD206^+^ (BD Biosciences) and CD86^+^ (BD Biosciences) populations among TAMs derived from sorted cells.

### Flow Cytometry

MΦs were processed into single-cell suspensions and blocked with 10% human AB serum (Sigma Aldrich) in PBS on ice. Then, the cells were stained with antibodies for 1 h at 4 °C in the dark. The cells were washed twice with 4 mL flow buffer, then centrifuged and resuspended in 0.5 mL flow buffer for analysis. Flow cytometry was performed using an ACEA NovoCyte Quanteon.

### In vitro cell proliferation, invasion/migration, and spheroid formation assays

Cell viability or growth was evaluated by a cell proliferation kit (XTT) (Roche). Briefly, cells were seeded in 96-well plates and treated with conditioned medium (CM) at a 1:3 ratio for different durations. A mixture of XTT reagents was added to each well and cultured with cells at 37 °C for 4 h. The absorbance at 492 nm was read. The relative cell viability is expressed as the fold change over the mean of the first day. Cell migration and invasion were determined using transwell cell migration assay kits (Corning, 8 μm) and BioCoat™ Matrigel^®^ Invasion Chambers (Corning, 8 μm), respectively. Cell samples (1 × 10^5^ cells) were briefly suspended in a serum-free medium and seeded in 24-well transwell chambers. Medium containing 1% FBS was added to the lower chamber (Supplementary Fig. 3A, *left*). The chamber was fixed with 100% methanol for 3 min and stained with 0.5% crystal violet (w/v) for 1 h. Three different fields of each chamber were photographed randomly using a microscope (ZEISS). For the spheroid assay, MΦs were seeded in the upper while EOC cells were in the lower chambers of a low-attachment plate (Supplementary Fig. 3A, *right*). As previously described [13], following a 48-hour stabilization period, the two cell types were co-cultured for a duration. Following this period, the spheroids that developed in the lower chamber were imaged. Then, the spheroid diameters were measured from two-dimensional images at the z-plane, where they presented a well-defined boundary. The quantification was done by manually counting the number of cells in a spheroid cross-section.

### Syngeneic mouse model of ovarian cancer

For ID8 syngeneic mouse model, homozygous *Yap1*^−/−^ and WT female mice (8–12 weeks old) were *i.p.* injected with a total of 1.0 × 10^7^ ID8 or ID8-GFP/Lucifer cells. After 14 days, tumors formed and were collected for flow cytometry and histological analysis. Tumor burden was estimated weekly by injecting the mice (*i.p.*) with 100 mg/kg D-Luciferin and potassium salt (Gold Biotechnology), followed by in vivo bioluminescence imaging using an IVIS Lumina X5 (PerkinElmer) until 49 days. Animals were euthanized during the whole-body imaging. The select tissues were excised for fluorescence quantification using the IVIS imaging system. Subsequently, the regions of interest (ROIs) within the tissues were delineated and quantified employing Living Image 4.0 software. WT mice received intraperitoneal (*i.p.*) injections of XMU-MP1 (1 mg/kg) (MOLNOVA) or a corresponding vehicle, both commencing at two weeks of age. For the immunofluorescence staining of spheroids derived from mice, an equal volume of ascites (500 µL) from each mouse was collected, which was then centrifuged to harvest cell spheroids. Then, a 40 μm mesh filter was employed to recover cell clusters in a reverse filtration process. These cells were subsequently utilized for immunofluorescence analysis, with most of the observed effects attributable to gene knockout. The entire animal study was performed according to guidelines approved by The Committee on the Use of Live Animals in Teaching and Research of The University of Hong Kong (CULATR number: 5489-20).

### Data collection and processing

The R package Seurat was applied to re-analyze the dataset GSE181935 [23]. Genes with the most significant changes were identified. Principal component analysis (PCAs) was performed, and the top 30 PCAs were used for dimensional reduction. The four final Seurat objects generated for each sample were integrated into a larger Seurat object through anchoring identification using the FindIntergationAnchors and IntergrateData Seurat functions. The cell clusters were found by FindClusters with resolution = 2. The FindAllMarkers were used in Seurat to perform the differential gene expression (DGE) analysis. The bulk RNA-seq data of ovarian cancer patients in TCGA-OV downloaded from the GDC data portal (http://portal.gdc.cancer.gov/) were used to evaluate the difference between the high and low gene expression groups.

### Statistical analysis

Experimental data are presented as the mean ± standard error mean (SEM) from at least three independent experiments. The results were analyzed with an unpaired t-test among two groups if both groups were normally distributed. Statistical analyses were performed using GraphPad Prism 8 (GraphPad Software). Differences were considered significant when the *p*-value was < 0.05. Comparisons among three or more groups were performed using one-way ANOVA and a multiple comparison test. (*, *p* < 0.05; **, *p* < 0.01; ***, *p* < 0.001).

## Results

### EOC peritoneal metastases are associated with protumoral TAM deposition

**Fig. 1:**
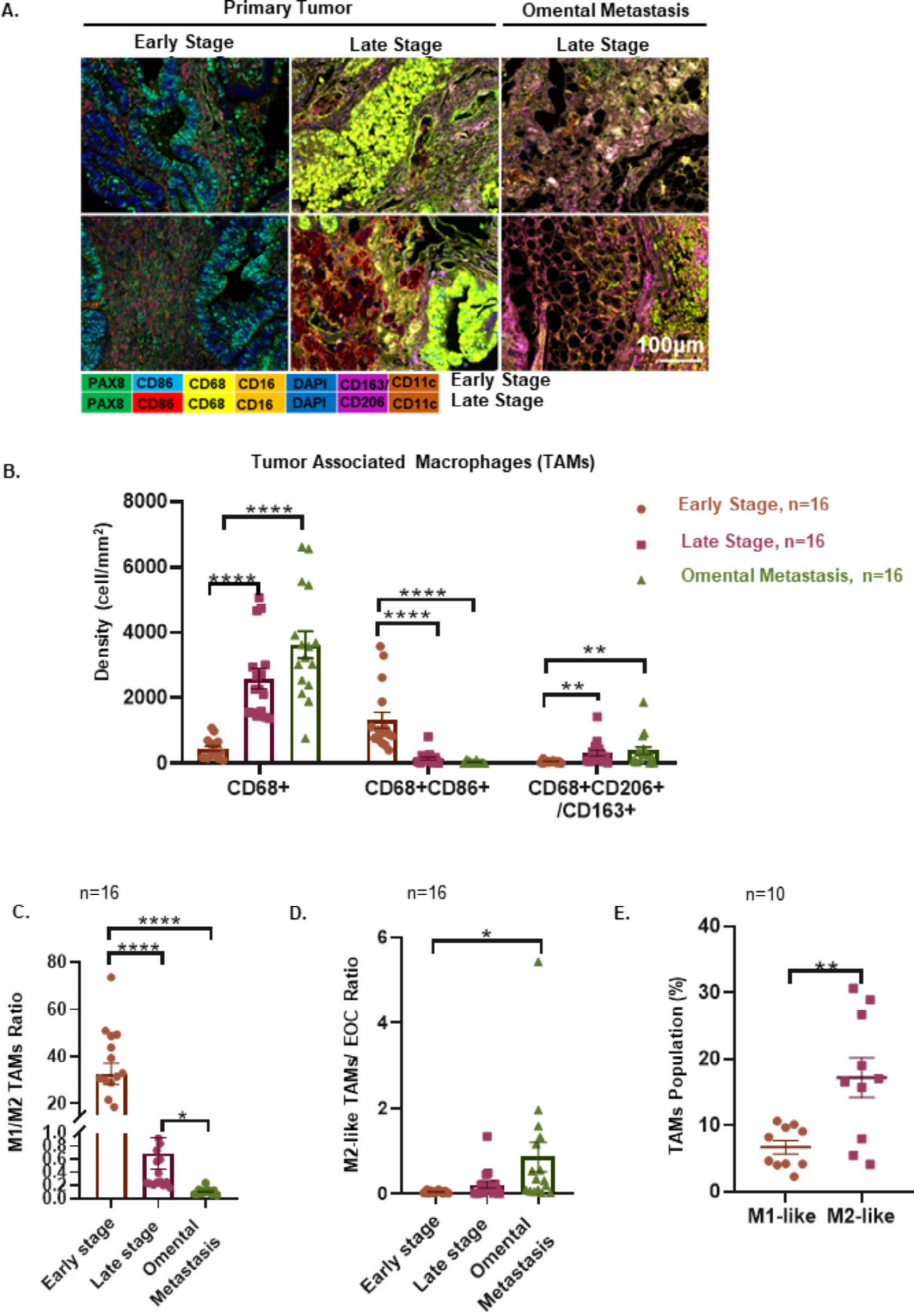
Malignant involvement of the serous body cavities is associated with converting M1-like TAMs to M2-like TAMs. (**A**) Representative composite of the m-IHC panel. Scale bar: 100 μm. (**B**) Distinct distributions of TAM population densities across regions in early stage (*n* = 16), late stage (*n* = 16), and omental metastasis (*n* = 16). (**C**) Ratio of M1-like polarized MФs (CD86^+^CD68^+^) to M2-like TAMs (CD206^+^/CD163^+^CD68^+^) (*n* = 16). (**D**) Ratio of M2-like TAMs to EOC (PAX8^+^) (*n* = 16). (**E**) Flow cytometric staining of CD11b^+^CD14^+^CD68^+^CD86^+^ (M1-like TAMs) or CD11b^+^CD14^+^CD68^+^CD206^+^ (M2-like TAMs) ascites TAMs from patients with EOC (*n* = 10). The data were presented as the means ± SEM and were analyzed by unpaired Student’s t-tests. *, *P* ≤ 0.05; **, *P* ≤ 0.01; ***, *P* ≤ 0.001; ****, *P* ≤ 0.0001

To understand the macrophages (MФs) diversity in EOC tumor progression, multiplex immunofluorescence (m-IHC) staining was employed to evaluate MΦs density and status in 32 EOC clinical samples, including primary tumors from early stages (I and II) to late stages (III and IV) and omental metastatic tumors (III and IV). MΦs were found in all tumor specimens and were significantly increased with tumor progression. The density of CD68^+^CD163^+^/CD206^+^ MΦs dramatically increased from the primary tumor at the early stage to omental metastases at the late stage during EOC progression (early stage to late stage *p* = 0.0055; early stage to omental metastasis *p* = 0.0091), whereas the density of CD68^+^CD86^+^ MΦs correspondingly decreased (early stage to late stage *p* < 0.0001; early stage to omental metastasis *p* < 0.0001), contributing to the shift in MΦs from CD86^+^ MΦs (M1-like) to CD163^+^/CD206^+^ (M2-like) phenotype (Fig. 1A and C; Supplementary Fig. S1A). Of note, the ratio of CD163^+^/CD206^+^ TAMs to ovarian cancer cells was increased in omental metastatic tumors (*p* = 0.0234), and the ratio of CD86^+^ TAMs to ovarian cancer cells was not (*p* = 0.3777), indicating that the metastatic microenvironment skews CD163^+^/CD206^+^ TAM polarization (Fig. 1D; Supplementary Fig. S1B). In addition, TAMs (CD206^+^CD45^+^CD11b^+^CD14^+^CD3^−^CD56^−^CD19^−^) infiltrated in primary tumors, paired omental metastatic tumors were compared, and the results confirmed the accumulation of CD206^+^ TAMs in the metastatic location (Supplementary Fig. S1C).

Ascites is a sign of peritoneal metastases, creating a prometastatic microenvironment and heralding a poor prognosis [24, 25]. To further assess TAM polarization during peritoneal metastases, the TAM population was evaluated in the ascites of EOC patients (*n* = 10). Similar to the TAM polarization in omental metastatic tissues, CD206^+^ TAMs (M2-like TAMs) were significantly increased when compared to CD86^+^ TAMs (M1-like TAMs) in ascites (Fig. 1E, *p* = 0.037; Supplementary Fig. S1D). Previous reports showed that TAMs are essential in ascitic tumor spheroid formation [26]. However, the roles of TAMs in the microenvironment of ascites spheroids of EOC remain ill-defined. Immunofluorescence assays identified EOC ascites spheroids as multicellular structures containing ovarian tumor cells (PAX8^+^Ki67^+^) and numerous lymphocytes, such as TAMs (CD68^+^CD206^+^, CD68^+^CD86^+^), T cells (CD8^+^), DCs (CD11c^+^) and NK cells (CD56^dim^, CD56^bright^) (Supplementary Fig. S1E). These clinical data suggest that protumoral M2-like TAMs accumulated in the peritoneum correlated with EOC peritoneal diffusion.

### OCM/AS skews protumoral TAM polarization

**Fig. 2:**
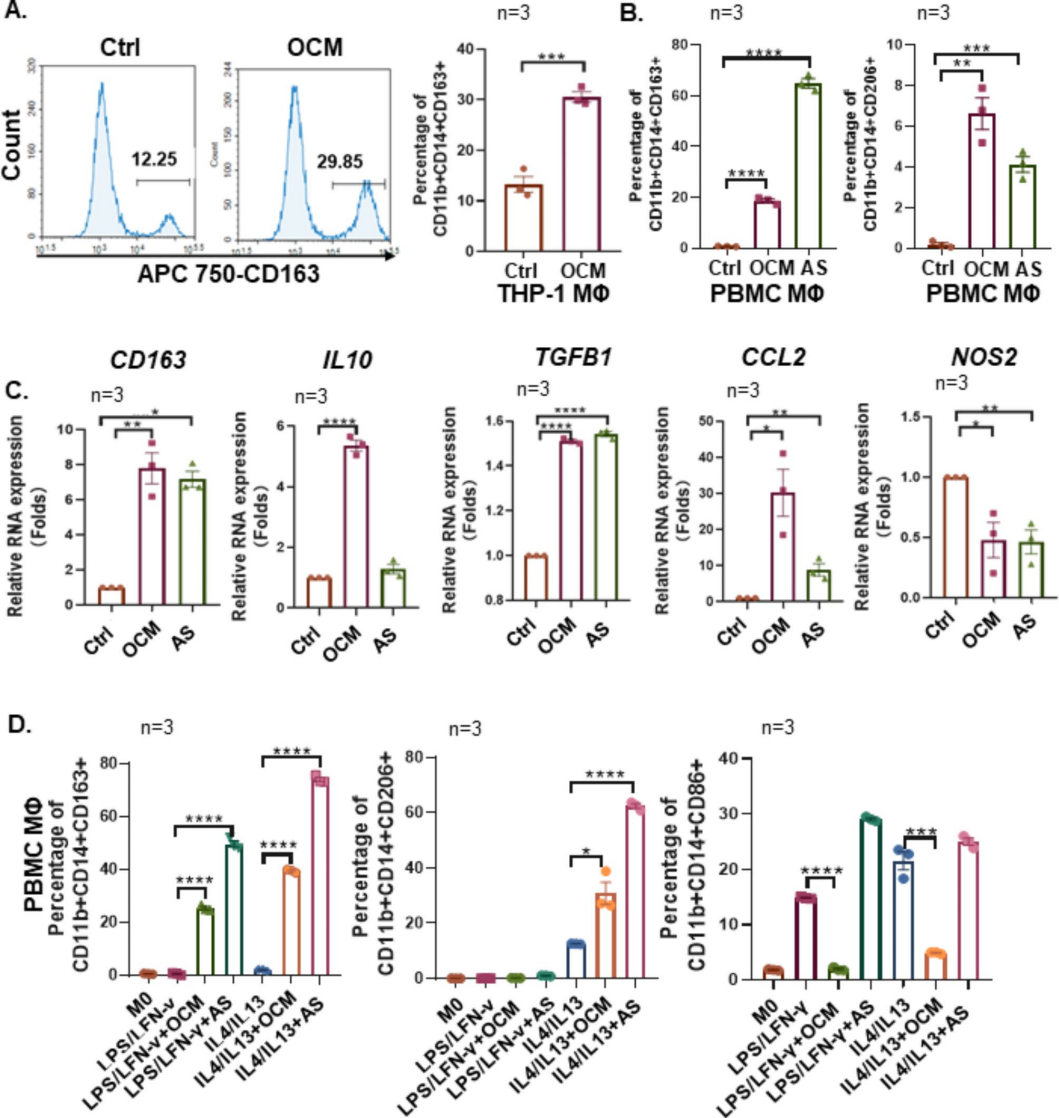
OCM/AS promotes M2-like TAMs polarization. (**A**) M0 MΦs (THP-1 MΦs, CD11b^+^CD14^+^) were treated with control medium or OCM and then, the expression of CD206 was analyzed by flow cytometry (*n* = 3). Representative flow cytometric plots (left) and summary bar chart (right). (**B**) M0 MФs (PBMC MΦs, CD11b^+^CD14^+^) were treated with OCM or ascites fluid (AS), and the expression of CD163 and CD206 was analyzed in OCM/AS treated macrophages or control macrophages by flow cytometry (*n* = 3). (**C**) The mRNA expression levels of *CD163*, *IL10*, *TGFβ*, *CCL2*, and *NOS2* in M0 MФs (PBMC MΦs) treated with OCM/AS or control medium were measured by qPCR. Gene expression data were normalized to the reference gene *18 S*, and the results are presented as the fold change relative to the control group. (**D**) M0 MФs (PBMC MΦs) were pre-stimulated with LPS/IFNγ (20 pg/mL, 20 ng/mL) to make M1 MΦs and were pre-stimulated with IL4/IL13 (20 ng/mL, 20 ng/mL) to make M2 MΦs. After that, MΦs (M1 & M2) were treated with OCM/AS or control medium, and then, the percentage of CD206, CD163, or CD86 were analyzed in macrophages and compared to M0 MΦs. The data were shown as the mean ± SEM and were analyzed by unpaired Student’s t-tests. *n* = 3, *, *P* ≤ 0.05; **, *P* ≤ 0.01; ***, *P* ≤ 0.001; ****, *P* ≤ 0.0001

Clinical evidence shows that M2-like TAMs frequently accumulate in ovarian cancer ascites and potentially be correlated with peritoneal metastases [1, 26]. To clarify these findings, FACS analysis and confocal microscopy analyses both found that the resting macrophages (M0 MΦs) derived from human peripheral blood mononuclear cells (PBMC MΦs) and THP-1 (THP-1 MΦs) pretreated with ascites fluid (AS), or omental conditioned medium (OCM) [12] showed a substantial increase in CD163^+^ and CD206^+^ expression levels (Fig. 2A and B; Supplementary Figs. S2A-2B, S2D). Consistently, qPCR analysis confirmed that the OCM/AS facilitated the expression of M2-like TAMs marker genes such as *CD163*, *IL-10*, *TGFβ1*, and *CCL2*, while downregulating the expression of the M1-like TAMs marker *NOS2* in MΦs (Fig. 2C, Supplementary Fig. S2C). Next, to verify whether the OCM/AS caused an imbalance in conventional M1/M2 polarization and skewed protumoral M2-like TAMs accumulation, M0 MΦs were activated to either the M1-like phenotype by human LPS/IFNγ or the M2-like phenotype by human IL4/IL13 upon OCM or AS treatment (Supplementary Fig. S2E). The results showed that the CD163^+^ population was increased after incubation in the OCM/AS regardless of prior treatment with LPS/IFNγ or IL4/IL13 (Fig. 2D). Similar enhancement occurred in IL4/IL13-stimulated CD206^+^ TAMs after culture in the OCM/AS, whereas a reduction in the CD86^+^ population occurred after OCM treatment, supporting the notion that the polarization of protumoral M2-like TAMs was induced by the OCM/AS (Fig. 2D). Taken together, these findings imply that there is a natural selection of choosing protumoral M2-like polarization in OCM/AS instead of M1-like polarization, which contribute to a higher ratio of M2- to M1-like phenotype MФs accumulation in malignant ascites.

### TAMs polarized in OCM/AS enhance the oncogenic potential in EOC cells

**Fig. 3:**
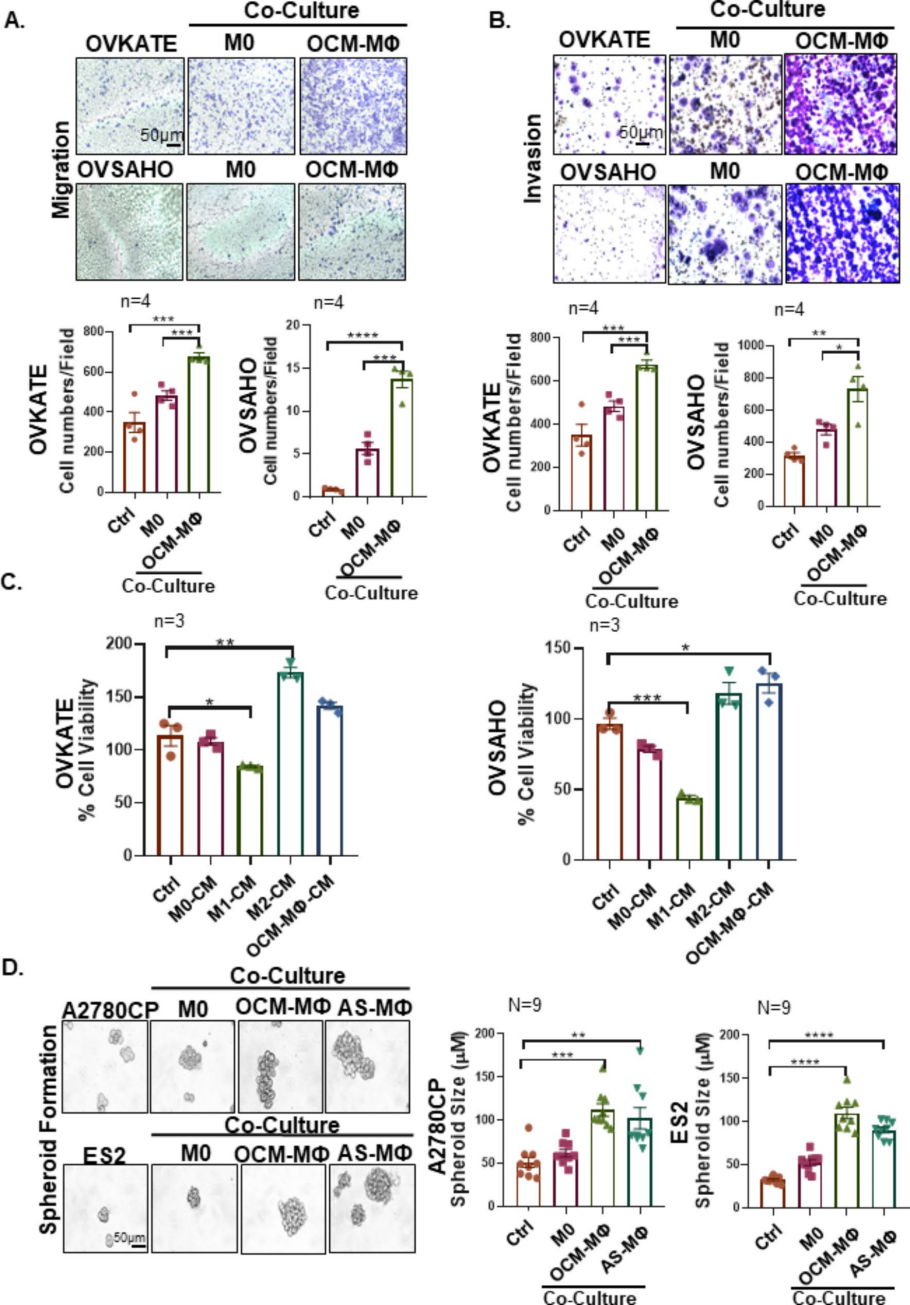
TAMs polarized in OCM/AS enhance the metastatic potential in EOC. (**A-D**) EOC cells were seeded in the upper chamber, while M0 MΦs, OCM-MΦs, or no cells were seeded in the lower chamber of the transwell plates and incubated for 36 h. The stained cells were counted in four randomly selected fields. Representative images and quantitative results of cell migration and invasion are shown (*n* = 4). Scale bar: 50 μm. (**E-F**) The ovarian cancer cell lines OVKATE and OVSAHO cells were incubated with either the control or the conditioned medium (CM) from various macrophage subsets (M0 MФs, OCM-MФs, M1 MФs, or M2 MФs). Subsequently, the viability of OVKATE and OVSAHO cells was assessed by the XTT cell proliferation assay. All the experiments were performed in triplicate (*n* = 3). (**G-H**) The spheroid formation capacity of EOC cells alone or co-cultured with control MΦs or TAMs was determined by spheroid formation assays. The size of the spheroids was quantified as shown (*n* = 9). Scale bar: 50 μm. The data were shown as the mean ± SEM and were analyzed by unpaired Student’s t-tests. The number of independent experiments was *n* = 9, *, *P* ≤ 0.05; **, *P* ≤ 0.01; ***, *P* ≤ 0.001; ****, *P* ≤ 0.0001

Transwell cell migration and invasion assays were conducted to determine the pro-tumoral function of TAMs polarized within the OCM/AS. These assays revealed that the human ovarian cancer cell lines exhibited enhanced migration and invasion capabilities when co-cultured with M0 macrophages pre-treated with OCM (Fig. 3A and D; Supplementary Fig. S3A, Fig. S3D-S3E). In contrast, only a slight increase in cell migration and invasion capacity was observed in the same EOC cells co-cultured with the resting M0 MΦs (Fig. 3A and D, Supplementary Fig. S3D-S3E). XTT cell proliferation assay revealed that the conditioned medium (CM) of M2 MΦs and OCM-MΦs markedly enhanced the proliferation and viability of EOC cells but it could not observe such increased cell proliferation in EOC cells when EOC cells co-cultured with the CM of the M0 MΦs (Fig. 3E F). Intriguingly, EOC cells co-cultured in the CM of M1 MΦs even significantly impaired cell growth (Fig. 3E F). Likewise, the spheroid formation assay demonstrated that the average sizes of spheroids formed from human ovarian cancer cell lines were significantly larger when co-cultured with OCM-MΦs and AS-MΦs than that with the respective M0 MΦs co-cultures and controls (Fig. 3G H; Supplementary Fig. S3B-S3C, Fig. S3F). These findings indicate that TAMs polarized in OCM/AS enhance the metastatic potential in EOC cells.

### Lipid metabolic activities increased in protumoral TAMs

**Fig. 4:**
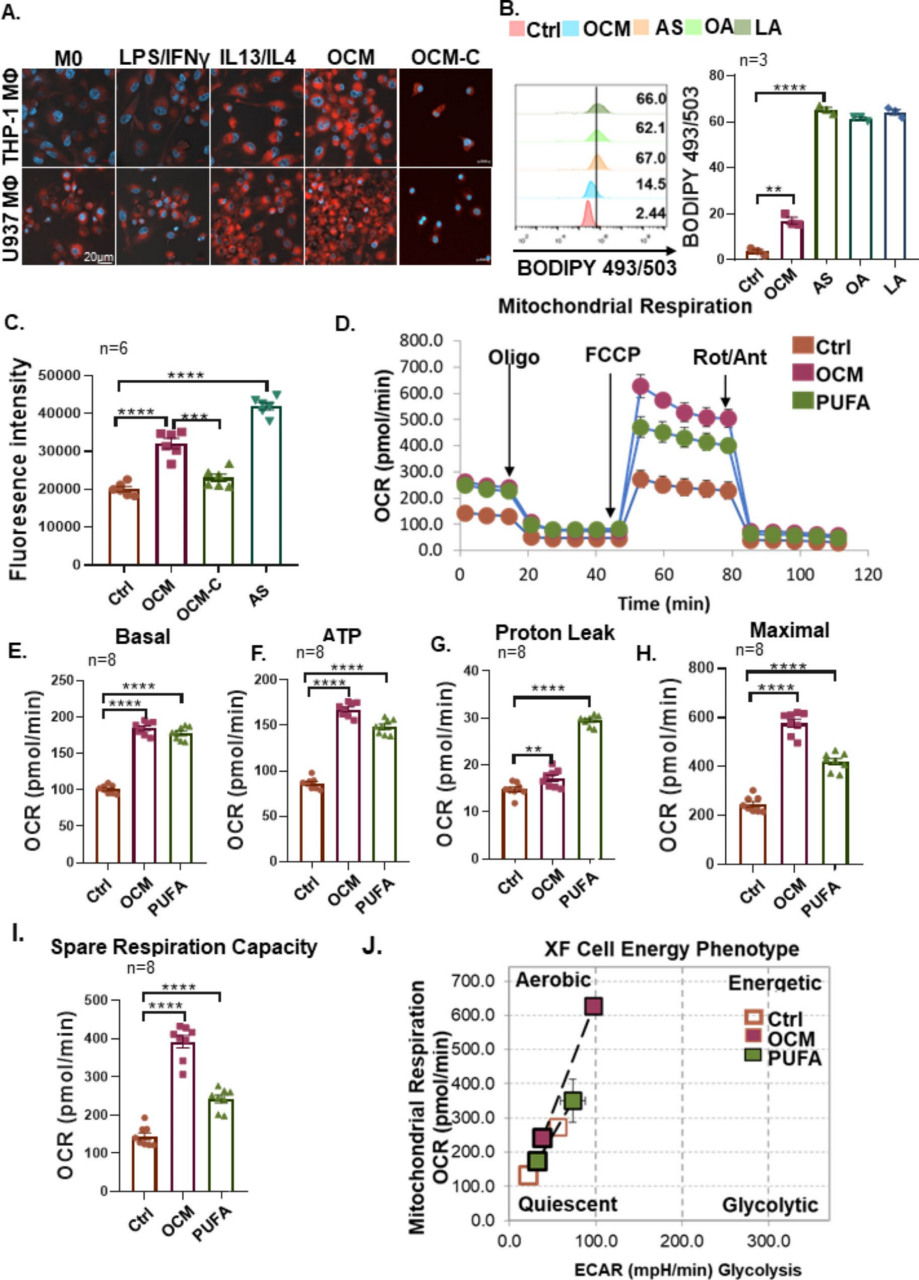
PUFAs-enriched OCM enhances lipid oxidation metabolism in TAMs. (**A**) The lipid droplet formation assay was measured using Nile Red staining. M0 MФs (THP-1 and U937 cells) treated with control medium, LPS/IFN-γ, IL4/IL13, OCM, or Cleanascite™ lipid removal reagent OCM were shown as OCM-C. Then, the different macrophages were stained with Nile Red and photographed by fluorescence microscope imaging. Representative pictures are shown in the figures. (**B** and **C**) The BODIPY fluorescent of M0 MФs (PBMC MФs) treated with OCM, ascites fluids (AS), oleic acid (OA, 200 µM), linoleic acid (LA, 200 µM) or control medium were analyzed by flow cytometry. The representative histogram was shown on the left, and statistical results of lipids’ mean fluorescence intensity (MFI) in MФs were shown in the right panel. (**D**) The fluorescence of reactive oxygen species (ROS) in M0 MФs (PBMC MФ) was measured by using DCFDA / H2DCFDA assay kit, where M0 was treated with control medium, OCM, ascites fluids (AS), or OCM. The statistical ROS fluorescence intensity was shown in the figure, *n* = 6. (**E**) Representative measurements of the OCR after the addition of oligomycin A (1 mM), FCCP (1.5 mM), and Rotenone and Antimycin (Rot/Ant) (0.5 mM) to M0 MΦs (THP-1 MФs) treated with control medium, OCM or PUFAs (linoleic acid, 200 µM). (**F**) Quantified basal OXPHOS. (**G**) Respiration-linked ATP. (**H**) Proton leakage. H^+^ (proton) leakage equals to (Maximum rate measurement after FCCP injection)-(Non-Mitochondrial Respiration). (**I**) Maximal OXPHOS. (**J**) Spare respiration capacity. (**K**) Representative plots and quantification of the ratio of oxidative (OCR) versus glycolytic (ECAR) metabolism at baseline (open squares) and after stress (filled squares). Metabolic Potential Equations: Stressed OCR (%)=(Stressed OCR)/(Baseline OCR) × 100; Stressed ECAR (%) = (Stressed ECAR)/(Baseline ECAR) x 100. *n* = 8. The data were shown as the mean ± SEM and analyzed by unpaired Student’s t-tests. ***, *P* ≤ 0.001; ****; *P* ≤ 0.0001

Recent studies have suggested that lipid accumulation and metabolism are associated with the differentiation and activation of protumoral TAMs [27–29]. Hence, we examined the lipid droplets (LDs) in MФs, which are cellular storage organelles for neutral lipids. Results showed that LDs significantly increased in the cytosol of IL4/IL13-induced M2 MΦs or OCM-MΦs but not in the cytoplasmic compartment of M0 MФs and M1 MΦs (LPS/INFγ) (Fig. 4A). Intriguingly, the removal of free fatty acids in OCM by the Cleanascite™ attenuated LDs deposition in OCM-C MФs (macrophages polarized in OCM pretreated with Cleanascite™) (Fig. 4A), indicating M2 MФs and OCM-MФs exhibited higher lipid accumulation and metabolism. To confirm the contribution of lipid accumulation on the polarization of protumoral TAMs, FACS analysis using the lipophilic fluorescent dye BODIPY 493/503 showed that OCM/AS-MФs displayed a significantly higher level of lipid accumulation (Fig. 4B and C). Our previous report has mentioned that intercellular unsaturated fatty acids (UFAs) are remarkably upregulated in ovarian cancer cells from the OCM/AS of EOC patients with peritoneal metastases [12, 13]. The lipidomic analysis further revealed that UFAs, such as linoleic acid (C18:2, LA) are abundant in ascites, and OCM and UFAs are highly uptake in ovarian cancer cells [12, 13]. Likewise, co-treatment with UFAs, such as linoleic acid (LA, 200µM) and oleic acid (OA, 200µM), could significantly increase the associated lipid contents within the M0 MΦs which was in line with what we observed in TAMs polarized in OCM/AS (Fig. 4B and C). A previous study demonstrated that high intercellular lipid contents in MФs accompanied by increased levels of cytosolic reactive oxygen species (ROS) are required for protumoral TAM polarization [30]. Likewise, we demonstrated that the cellular ROS levels in OCM/AS-MΦs derived from PBMC MΦs were significantly upregulated, whereas the addition of Cleanascite™ mitigated the increased ascites-mediated ROS level, indicating that the accumulation of intercellular UFAs is responsible for the enhanced ROS production in OCM-MФs (Fig. 4D).

### M2-like TAMs polarized in OCM/AS exhibit enhanced lipids-OXPHOS

The TME is a metabolically challenging environment for tumor growth and progression. Alterations in the nutrients and metabolic signals of the TME could highly intervene in TAMs and reprogram their functions. Generally, M1 MФs run on aerobic glycolysis, whereas M2 MФs rely on mitochondrial OXPHOS [31, 32]. To confirm whether M1 MФs and M2 MΦs display distinct metabolic patterns as above, we first assessed the cellular bioenergetics of the M1 and M2 MФs derived from mouse bone marrow-derived MΦs (BMDMs). The mitochondrial respiratory profile showed that M2 MΦs exhibited increased mitochondrial respiration (similar to OCM-MΦs) as compared with M0 MФs and M1 MФs (Supplementary Fig. S4A). In contrast, M1 MΦs exhibited impaired mitochondrial function, as indicated by a lower basal OCR and a lack of response to FCCP (Supplementary Figs. S4B-4C). Given that both lipid accumulation and metabolism are associated with the differentiation and activation of protumoral TAMs [29], the live-cell oxygen consumption rate (OCR) together with extracellular acidification rate (ECAR) were applied to characterize the cellular respiratory profile of TAMs in malignant ascites. Previously, we and others have indicated that the acellular fraction of OCM/AS is enriched with free fatty acids, particularly unsaturated fatty acids (UFAs) [12, 33]. As expected, analyses of basal and ATP-dependent oxygen consumption rate (OCR) revealed that both omental conditional medium (OCM) and polyunsaturated fatty acid (PUFA), specifically linoleic acid (LA), significantly elevated OCR in M0 MΦs. This suggests that UFAs, particularly the predominant PUFA, LA, may promote M2-like polarization (Fig. 4E and G). Most of the basal mitochondrial OCR was attributed to ATP production, as indicated by the suppression of oligomycin A (1 µM) (Fig. 4F). Any remaining OCR was likely due to proton leakage across the membrane (Fig. 4H), similar to that in the OCM and control groups. In addition, maximal OCR (Fig. 4I) and respiratory reserve capacity both were significantly increased in OCM-MФs or MФs treated with PUFA (Fig. 4I and J), confirming that oxidative phosphorylation respiration was elevated in TAM polarization within OCM/PUFA. Besides, to determine the bioenergetic categories that TAMs fall into upon OCM/PUFA education, the bioenergetic profiles of MФs within different treatments were also plotted. The results showed that OCM MΦs and MФs treated with PUFA were more energetic and highly aerobic than M0 MФs (Fig. 4K), augmenting oxidative phosphorylation in TAMs polarized in lipid-enriched OCM. Collectively, these results suggest that malignant ascites-derived PUFAs are associated with metabolic plasticity, particularly in M2-like TAMs.

### PUFAs promote protumoral TAM polarization via the RhoA-YAP1 cascade

**Fig. 5:**
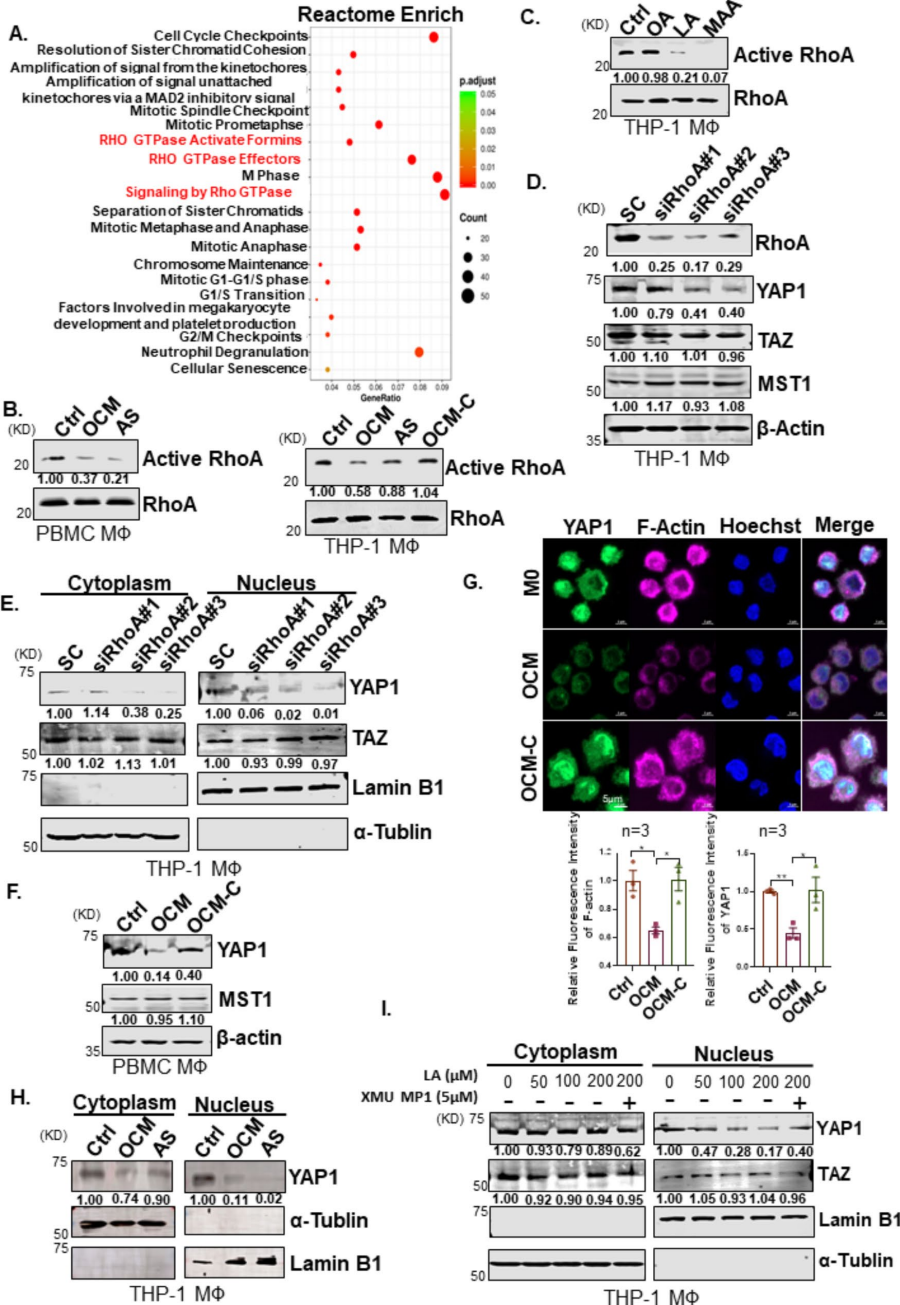
TAMs polarized in OCM/AS via inactivating RhoA-YAP1 signaling cascade. (**A**) M0 MФs (THP-1) were stimulated with LPS/LFN-γ to make M1 MФs. After that, M1 MФs were treated with OCM or a control medium. RNA-seq analysis compared the OCM-treated M1 MФs versus non-OCM-treated M1 MФs. Functional enrichment and pathway analysis of significantly differentially expressed mRNAs. The results show the enriched related pathways. (**B** and **C**) Pull-down assays for the active RhoA in OCM-MФs, AS-MФs (PBMC MΦs (upper) or THP-1 MΦs (lower)). (**D**) Linoleic acid (LA, 200 µM) and methyl arachidonate (MAA, 200 µM), but not oleic acid (OA, 200 µM), led to a reduction in RhoA activity in M0 MФs (PBMC) after 24 h treatment. (**E**) Knockdown of RhoA is confirmed by Western blotting. After transfecting M0 MΦs (THP-1 MФs) by RhoA siRNAs (#1 - #3) or scrambled siRNA (SC) for 48 h, cellular proteins were collected, and RhoA, YAP1, TAZ, MST1, and β-actin were detected by immunoblotting. (**F**) The localization of YAP and TAZ in the cytoplasm and nuclear was measured in MФs cells after 48 h post-transfection of RhoA siRNAs (#1 - #3) or scrambled siRNA (SC). (**G**) The protein expression of YAP1 and MST1 were measured in M0 MФs (PBMC MФs) treated with the control medium, OCM, or OCM-C. The expression of β-actin was used as the internal control. (**H**) Immunofluorescence analyzed the expression of F-actin (pink) and YAP1 (green) in M0 MΦs treated with the control medium. Nuclei were counterstained with Hoechst (blue). (**I**) The results of relative F-actin and YAP1 expression in macrophages are shown in the figures. (*n* = 3). Scale bar: 5 μm. (**J**) The localization of YAP1 in the cytoplasm and nuclear was measured in M0 MΦs via western blot after OCM stimulation for 24 h. (**K**) Linoleic acid (LA) reduced nuclear YAP1 activity in a dose-dependent in MΦs after 24 h treatment. XMU MP1 (5µM) restores nuclear YAP1 in LA-treated MΦs

To elucidate the molecular mechanisms that promote the polarization of TAMs within OCM, transcriptomics analysis of M1 or M2 MФs exposed to OCM or a control medium was performed. Reactome pathway analysis revealed the significant downregulation in Rho-GTPase pathways in these OCM-educated MΦs (Fig. 5A; Supplementary Figs. S5A-5B). Notably, M1 and M2 MФs treated with OCM had significantly decreased expression of Rho-GTPase signaling factors compared with corresponding control MФs (Fig. 5A; Supplementary Figs. S5A-5B), suggesting a reciprocal relationship between Rho-GTPase transduction and the OCM education. Among the Rho family members, the small GTPase RhoA plays a central role in Rho-GTPase signaling, participating in numerous cellular processes and contributing to immune cell differentiation and function [34, 35]. To verify whether OCM downregulates Rho-GTPase activity in TAMs, the G-LISA RhoA activation and active RhoA pull-down analyses revealed that OCM significantly downregulated active RhoA in OCM/AS-MΦs (PBMC-MΦs and THP-1 MΦs) (Fig. 5B and C; Supplementary Fig. S6A). Given that UFAs are the main lipids elevated in ovarian cancer cells co-cultured with OCM/AS [12, 13, 36], we evaluated MUFA such as oleic acid (OA), and PUFAs such as linoleic acid (LA) and methyl arachidonate (MAA), the abundant UFAs in protumoral M2-like TAM polarization. Of note, instead of OA, both LA and MAA could markedly inhibit RhoA activity in MФs, suggesting that PUFAs in OCM/AS promote protumoral TAM polarization through RhoA-GTPase inhibition (Fig. 5D).

The Hippo-YAP/TAZ signaling pathway has a complex role in regulating cancer development depending on the specific cell types in tumor mass, especially in immune cells [37]. Evidence suggests that Rho-GTPase is closely involved in regulating YAP/TAZ transcriptional activities, and RhoA could positively regulate the YAP1 activities through F-actin cytoskeleton [38]. Consistently, our study found that the depletion of RhoA by the siRNA knockdown approach displayed a significant reduction of nuclear YAP1 but not TAZ in MΦs (Fig. 5E and F), implying that RhoA solely downregulates YAP1 expression in TAMs. Given that OCM/AS could suppress active RhoA in MΦs (Fig. 5B C; Supplementary Fig. 6A), we have thus confirmed that the lipids within OCM/AS could reduce the total and nuclear YAP1 expression in MΦs (Fig. 5F and J; Supplementary Figs. S6B-6I). In contrast, removing lipids by Cleanascite™ remarkably prevented the reduction of YAP1 in OCM-MΦs (Fig. 5G). Confocal images showed that the F-actin and YAP1 levels in OCM-treated MФs were lower than those in vehicle-treated cells, indicating that OCM could regulate cytoskeletal assembly (Fig. 5H and I; Supplementary Fig S5C). These findings suggest that the lipids within OCM/AS facilitate protumoral M2-like TAM polarization by negatively regulating the RhoA-YAP1 signaling axis. To further support this observation, we found that TAMs polarized in OCM/AS could reduce nuclear YAP1 (Fig. 5J; Supplementary Figs. S6G-6I). Likewise, two dominant PUFAs, LA and MAA, showed consistent findings, which could significantly reduce nuclear YAP1 expression but increase the CD163^+^ MФs population (Fig. 5K; Supplementary Figs. S6D-6F). MST1-YAP is a conserved Hippo pathway, and the activated MST1 could phosphorylate YAP1, leading to the degradation of YAP1 [39]. However, we observed that MST1 expression was not changed in OCM-MФs or PUFAs educated MФs, and also in MФs upon RhoA-siRNA suppression (Fig. 5E and F; Supplementary Fig. S6D). This indicates that the downregulation of YAP1 in OCM-derived TAMs by RhoA-YAP1 signaling regulatory pathway parallels that of the Hippo MST1/2-YAP1 pathway. Collectively, these data demonstrated that PUFAs-enriched OCM/AS suppress RhoA-YAP1 signaling through MST1/2-YAP1 independent cascade that, in turn, promotes protumoral M2-like TAM polarization.

### Loss of YAP1 is essential for M2-like TAM polarization

**Fig. 6:**
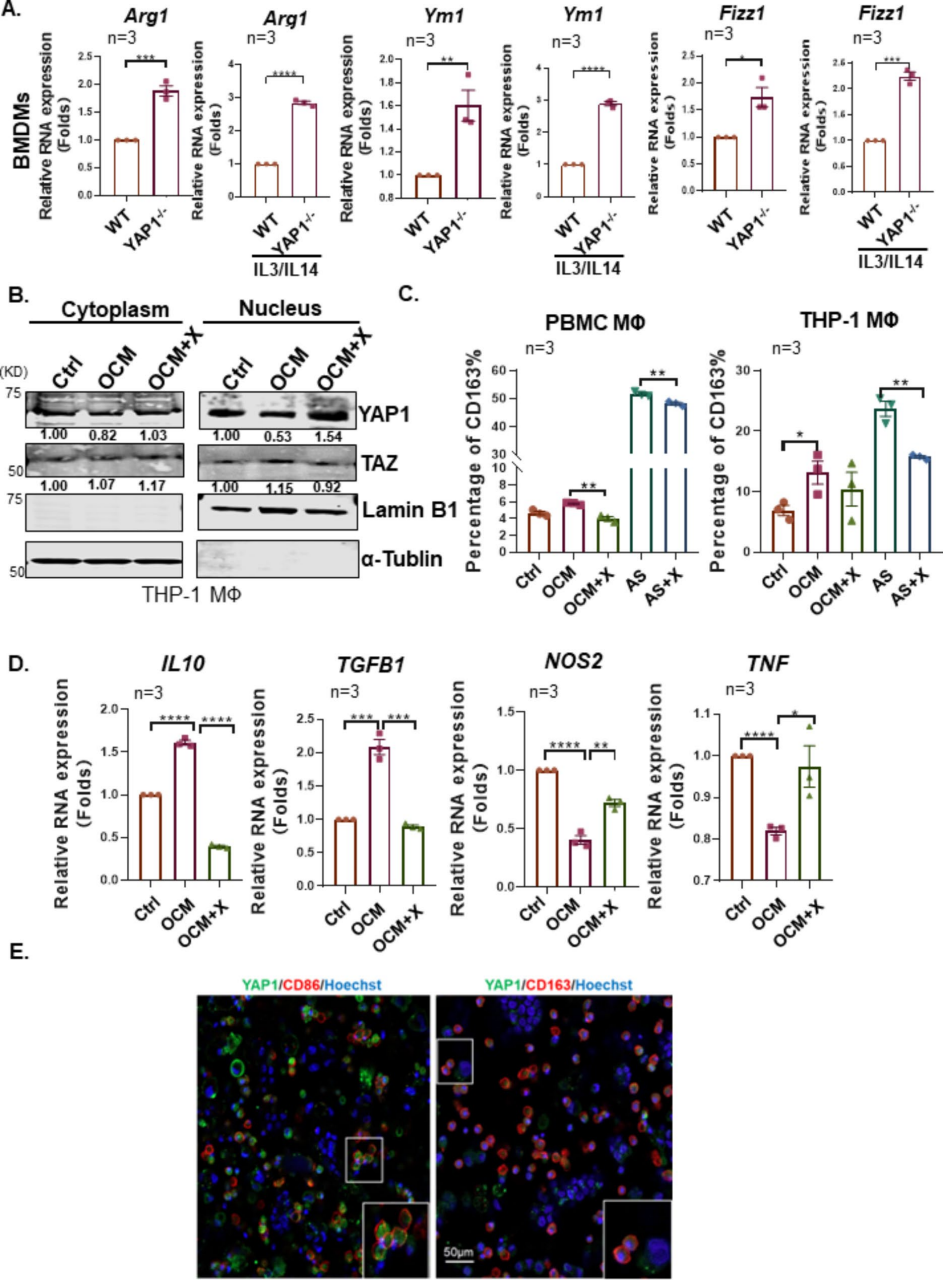
Inhibition of MST1/2 Activates YAP1 while impeding M2-like macrophage polarization. (**A**) WT and *Yap1*^−/−^ BMDMs were stimulated with murine IL-4/IL-13 (10 ng/mL, 10 ng/mL) or Ctrl for 24 h, and the mRNA levels of *Arg1*, *Fizz1*, and *Ym1* were detected by qPCR. Gene expression data were normalized to the reference gene *Gapdh*, and the results are presented as the fold change relative to the control. (**B**) The localization of YAP1 and TAZ in the cytoplasm and nuclear was measured in OCM-MФs or OCM-MФs treated with XMU MP1 (5 µM) for 24 h. (**C**) Comparison of the population of CD206 or CD163 in OCM-MФs, AS-MФs, and XMU MP1(5 µM) treated OCM/AS-MФs. (**D**) The mRNA levels of *IL10*, *TGFβ*, *NOS2*, and *TNF* in M0 MФs, OCM-MФs, or OCM-MФs treated with XMU MP1 (5 µM) were detected by qRT–PCR. Gene expression data were normalized to the reference gene *18 S*, and the results were presented as the fold change relative to the control treatment. (**E**) Immunofluorescent staining of YAP1^+^CD163^+^ and YAP1^−^CD86^+^ cells in ascites spheroids derived from EOC patients (*n* = 3). Hoechst was used to stain nuclei. Scale bar: 50 μm. The data were shown as the mean ± SEM and were analyzed by unpaired Student’s t-tests. *, *P* ≤ 0.05; **, *P* ≤ 0.01; ***, *P* ≤ 0.001; ****, *P* ≤ 0.0001

To confirm the causative role of YAP1 in MФs polarization, we adopted the siRNA knockdown to reduce the endogenous YAP1 in M0 MΦs. Results showed that the depletion of YAP1 led to the upregulation of M2-like associated markers such as *IL10*, *TGFβ*, and *CD163*, but the downregulation of M1-like associated markers such as *NOS2* in M0 MΦs (Supplementary Fig. S7A), indicating the loss of YAP1 favors M2-like MФs polarization. Further study by specifical deletion of the endogenous *Yap1* gene in myeloid lineage cells through the crossbreeding with Yap1^fl/fl^ and LysM^cre^ mice to generate Yap1 conditional knockout (cKO *Yap1*^*−/−*^) mice would be conducted (Supplementary Figs. S7B-7C). Compared to the WT control, the mouse bone marrow-derived MΦs (BMDMs) from Yap1 cKO (*Yap1*^*−/−*^) mice displayed the significant upregulation of M2-like MΦs associated genes such as *Arg1*, *Ym1*, and *Fizz1* (Fig. 6A). Likewise, *Yap1*^*−/−*^ MФs pretreated with murine IL4/IL13 showed the significant upregulation of M2-like MΦs associated genes such as *Arg1*, *Ym1*, and *Fizz1* compared with WT MФs pretreated with murine IL4/IL13 (Fig. 6A). Moreover, we analyzed the Cancer Genome Atlas Ovarian Cancer (TCGA-OV) database, and found CD68^+^YAP1^low^ groups exhibited dramatically increased M2-like TAMs markers expression, such as *MMP9*, *CCL8*, *SPP1* and *CCL17*, when compared to CD68^+^ YAP1^high^ (Supplementary Fig. S7D). Of note, the density of CD68^+^YAP1^high^ MΦs dramatically decreased from the primary EOC tumor at the early stage to omental metastases in advanced ovarian cancers (Supplementary Fig. S7E). In addition, TAMs isolated from the omental metastatic sites showed a significant reduction of *YAP1* mRNA expression compared to primary TAMs (Supplementary Fig. S7F). These data remarkably support our notion that the reduced YAP1 expression is a prerequisite for M2-like TAM polarization.

### Restoration of YAP1 by MST1/2 blockade inhibited M2-like TAM polarization

We next investigated whether the restoration of YAP1 could prevent M2-like TAM polarization. As expected, pharmacological suppression of MST1/2 by treatment of XMU MP1 efficiently restored YAP1 expression and activity [40]. To this end, the subcellular fractionation assay displayed that the nuclear YAP1, but not TAZ, in LA or OCM-educated MΦs (LA-MΦs/OCM-MΦs) could be restored by XMU MP1 (5µM) co-treatment (Figs. 5I and 6B). Immunofluorescence analysis also further showed the restoration of nuclear YAP1 upon treatment of XMU MP1 in OCM-MФs or AS-MФs (Supplementary Fig. S8A). Noticeably, the treatment of XMU MP1 considerably reduced the CD163^+^ population in OCM/AS-MΦs by downregulation of M2-like MΦs associated genes such as *IL10* and *TGFβ1* (Fig. 6C and D; Supplementary Fig. S8B). Intriguingly, the expressions of M1-like MΦs marker genes, such as *NOS2* and *TNF* were upregulated in OCM-MФs upon treatment of XMU MP1 (Fig. 6D), suggesting that the restoration of YAP1 by XMU MP1 could cause a conversion of M2- to M1-like MФs polarization.

To better understand the functional role of XMU MP1 in TAMs, the cellular respiratory profile of MΦs polarized in AS was characterized. Basal and ATP-dependent OCR readings indicated that AS-MΦs consistently exhibited a higher OCR than the untreated control, while this AS-mediated increase in OCR was apparently hampered by treatment with XMU MP1 (Supplementary Fig. S8C). The AS-induced accumulation of lipid droplets was significantly diminished upon treatment with XMU MP1 (Supplementary Fig. S8D). These findings manifest that XMU MP1-treated TAMs were at submaximal respiratory capacity, and the capacity of lipid droplet formation was impaired. The consistency is that CD163^+^ TAMs in ascites spheroids derived from EOC patients displayed nuclear YAP1 deficiency in contrast to CD86^+^ TAMs (Fig. 6E). These results manifested that the PUFAs-enriched ascites microenvironment promotes M2-like TAM polarization via the RhoA-YAP1 cascade deficiency. In contrast, restoring YAP1 expression by suppressing MST1/2 promotes M2-like TAMs to M1-like TAMs.

### YAP1 deficiency promotes protumoral M2-like TAM polarization in vivo

**Fig. 7:**
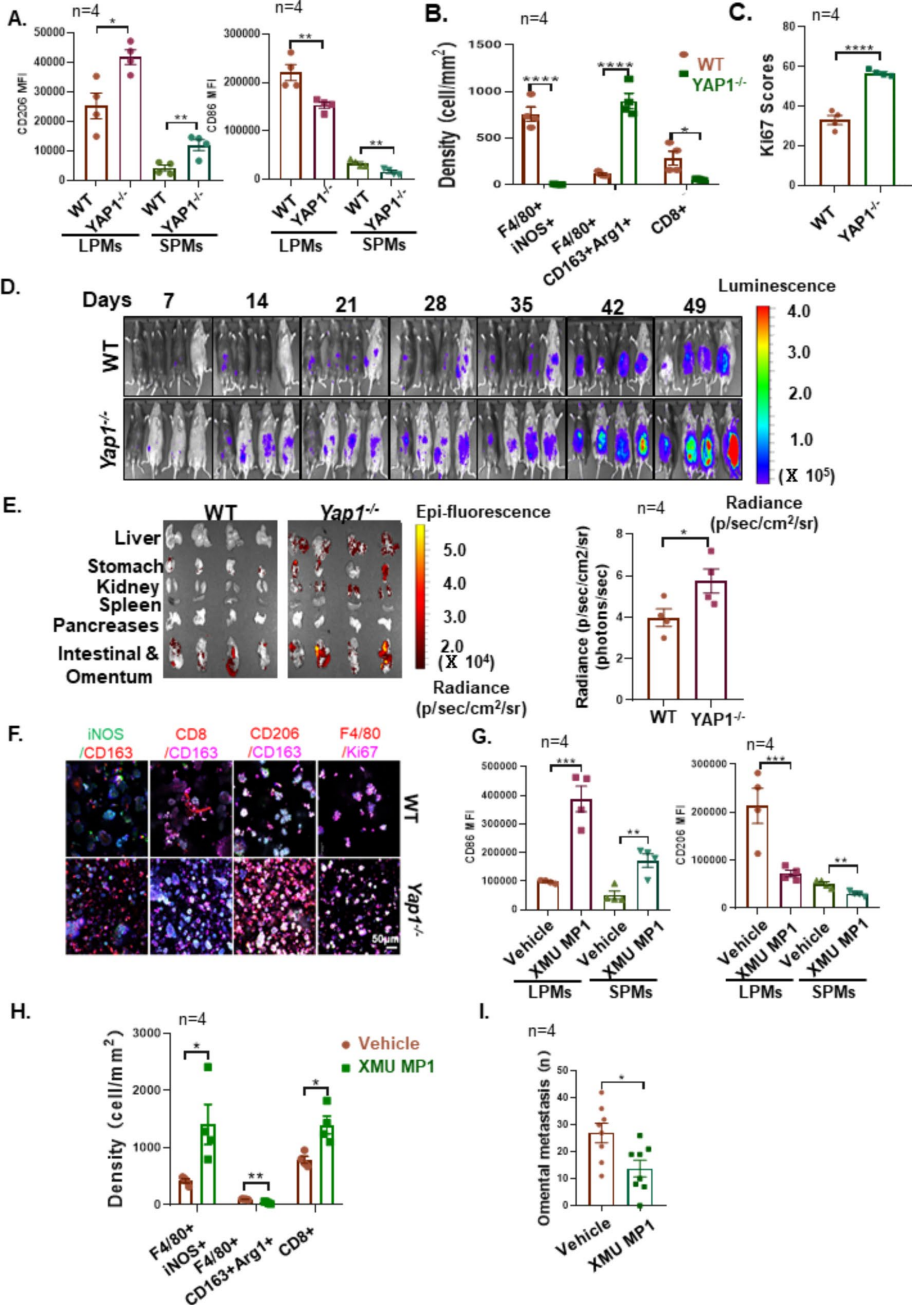
Abolishing Yap1 in MΦs results in an immunosuppressive microenvironment that facilitates EOC peritoneal metastases. (**A**) Quantification of the CD86^+^ and CD206^+^ mean of fluorescence intensity (MFI) in the LPMs and SPMs of WT and *Yap1*^−/−^ mice (*n* = 4) after 28 days’ intraperitoneal (*i.p.*) injection of ID8 cells. (**B**) Distinct distributions of iNOS^+^F4/80^+^, F4/80^+^CD206^+^Arg1^+^ population densities and infiltrating CD8^+^ T cells in WT and *Yap1*^−/−^ mice after 28 days *i.p.* injection of ID8 cells. (**C**) Ki67 expression was evaluated in omental isolated from WT and *Yap1*^−/−^ mice after 28 days *i.p.*, injection of ID8 cells. (**D**) Metastasis in WT and *Yap1*^−/−^ mice following *i.p.* injection was evaluated every 7 days by bioluminescence imaging. (**E**) Quantitative fluorescent analysis is calculated as the total fluorescence intensity per organ within the peritoneum post-49 days (left). The results of fluorescence quantification are presented on the right. (**F**) For the immunofluorescence staining, an equal volume of ascites (500 µL) from each mouse was collected and then centrifuged to harvest cell spheroids. Then, a 40 μm mesh filter was employed to recover cell clusters in reverse filtration. These cells were subsequently utilized for immunofluorescent staining of Ki67^+^F4/80^+,^ CD206^+^CD163^+^, CD8^+^CD163^+^, and iNOS^+^CD163^+^ cells in ascites spheroids from WT and *Yap1*^−/−^ mice (*n* = 4) after 49 days. Scale bar: 50 μm. (**G**) Quantification of the CD86^+^ and CD206^+^ MFI in peritoneal MФs (LPMs and SPMs) isolated from tumor-bearing mice (*i.p.* injection of ID8 cells) that were treated with vehicle or XMU MP1 (*n* = 4) for 28 days. (**H**) Distinct distributions of iNOS^+^F4/80^+^, F4/80^+^CD206^+^Arg1^+^ population densities and infiltrating CD8^+^ T cells in omental were evaluated by m-IHC after vehicle or XMU MP1 for 28-day treatment. (**I**) Quantification of omental metastases number (*n* = 4). The data were shown as the mean ± SEM and were analyzed by unpaired Student’s t-tests. *, *P* ≤ 0.05; **, *P* ≤ 0.01; ****, *P* ≤ 0.0001

To further assess the impact of Yap1 deficiency on protumoral M2-like TAM accumulation in vivo, the murine ovarian cancer cell line ID8 cells were intraperitoneally (*i.p.*) injected into the WT control and *Yap1*^−/−^ mice. After 28 days, FACS analysis was performed on two subsets of peritoneal macrophages (PEMs), large peritoneal MΦs (LPMs) and small peritoneal MΦs (SPMs), which were respectively defined as MHCII^−^/F4/80^+^ cells and MHCII^+^/F4/80^−^ cells in the murine peritoneal cavity [41]. Results showed that CD86^+^ MФs were reduced among both LPMs and SPMs in *Yap1*^−/−^ mice, while CD206^+^ MФs were increased among LPMs in *Yap1*^−/−^ mice compared with WT mice (Fig. 7A; Supplementary Figs. S9A-9B), suggesting that the Yap1 deficiency in TAMs promotes a protumoral microenvironment.

To study the contribution of Yap1 deficiency in MΦs on the pre-metastatic niche, omental tissues from WT and *Yap1*^−/−^ mice were analyzed by m-IHC. Results revealed that *Yap1*^−/−^ mice exhibited a significant reduction in F4/80^+^iNOS^+^ MФs and cytotoxic CD8^+^ T-cell infiltration, whereas the population of F4/80^+^CD206^+^Arg1^+^ MФs was significantly increased in *Yap1*^−/−^ mice compared with WT mice (Fig. 7B; Supplementary Fig. S9C). Moreover, Yap1 deficiency in MΦs is also associated with the increased tumor cell proliferation index (Ki67) in the omental metastatic tumor zone (Fig. 7C; Supplementary Fig. S9C), suggesting the loss of Yap1 contributes to the enhanced tumor growth. To investigate the impact of Yap1 deficiency on macrophage (MФ) polarization within the setting of epithelial ovarian cancer (EOC) peritoneal metastases, ID8 cells were genetically engineered to express the MSCV-GFP-T2A-Luciferase plasmid. These cells were then stably transfected and subsequently introduced into the peritoneal cavity of both WT and *Yap1*^−/−^ mice via intraperitoneal injection. After approximately two weeks, disseminated tumor cells were concomitantly detectable, and *Yap1*^−/−^ mice displayed a higher tumor growth rate than WT mice (Fig. 7D; Supplementary Fig. S9D). After 49 days’ injection, *Yap1*^−/−^ mice demonstrated a significant escalation in peritoneal metastases relative to WT mice, implying that Yap1 deficiency enhances macrophage-mediated peritoneal metastatic progression (Fig. 7E). Consistent with the m-IHC results shown in Fig. 7B, tumor spheroids formed by ascites in *Yap1*^−/−^ mice exhibited more CD206^+^CD163^+^ TAMs and fewer iNOS^+^CD163^+^ TAMs than the spheroids from WT mice (Fig. 7F). Furthermore, cytotoxic CD8^+^ T cells in the tumor spheroids of *Yap1*^−/−^ mice also decreased compared to those in WT mice (Fig. 7F).

### Restoration of YAP1 by targeting MST1/2 elevates M1-like polarized MΦs

Following the promising in vitro results where MST1/2 inhibition reversed M2-like protumoral polarization in TAMs, we further explored whether MST1/2 targeting could enhance the in vivo accumulation of tumoricidal M1-like polarized MФs. To do so, we intraperitoneally injected ID8 cells into WT mice. After one week, the mice were treated with vehicle or XMU MP1 (1 mg/kg) once every other day for 28 days. Peritoneal MΦs were then assessed by FACS analysis, and the results showed that CD86^+^ MФs (LPMs and SPMs) were significantly increased, but CD206^+^ MФs (LPMs and SPMs) were decreased in XMU MP1-treated WT mice without apparent toxicity (Fig. 7G; Supplementary Figs. S10A-S10C). On the other hand, the omental tissue was evaluated by m-IHC, and the results showed that iNOS^+^ MФs, infiltrated cytotoxic CD8^+^ T cells were significantly increased, and CD206^+^Arg1^+^MФs were reduced in XMU MP1 treated group (Fig. 7H; Supplementary Fig. S10B). XMU MP1-treated WT mice significantly reduced omental metastases and tumor growth compared with untreated WT mice (Fig. 7I; Supplementary Fig. S10D-10G). To further confirm the effect of MST1/2 depletion on MФs polarization, we assessed the differential gene expressions among the immune cells using the public scRNA data in LysMCre-Mst1/2^flox/flox^ (MST1/2^−/−^) and Mst1/2^flox/flox^ (WT) mice (GSE181935) (Supplementary Fig. S10H). As expected, results showed that the M2-like MФs marker genes *CD163*, *CD209a*, *Hdc*, *MS4a4b*, *Ptgds*, and *Snca* were downregulated in MST1/2^−/−^ mice compared to WT mice (Supplementary Fig. S10I). Taken together, these findings support the notion that the loss of YAP1 in MΦs accelerates EOC peritoneal metastases with M2-like TAM accumulation. In contrast, suppressing MST1/2 by XMU MP1 could elevate M1-like polarized MФs and cytotoxic CD8^+^ T cells population, attenuating overt peritoneal metastases of EOC.

## Discussion

The TME plays a critical role in tumor growth and metastatic progression [42], yet the underlying mechanisms of TME in reprogramming the extremely plastic TAMs to the protumoral M2-like TAMs remain unclear. In this study, we add a novel insight into the impact of lipid-enriched OCM/AS on controlling tumor immunogenicity through accumulating protumoral M2-like TAMs from tumor-infiltrating MФs in peritoneal metastases of EOC. Our validity evidence showed that enriched PUFAs in malignant ascites activated lipid-OXPHOS metabolism in tumor-infiltrating MФs, skewing protumoral M2-like TAM polarization by coordinating the RhoA-YAP1 signaling cascade. Noticeably, YAP1 deficiency due to PUFAs-mediated downregulation of RhoA in MФs drives protumoral M2-like TAM polarization that, in turn, attenuates cytotoxic CD8^+^ T cell infiltration, promoting metastatic progression. In contrast, targeted suppression of MST1/2 (Hippo) with the specific inhibitor, XMU MP1, elevates nuclear YAP1 expression and encourages the conversion of M2- to M1-like phenotypes of TAMs that leads to the restoration of cytotoxic CD8^+^ T cells recruitment and activity, which in turn, inhibited EOC metastatic progression (Supplementary Fig. S11). These findings demystify the longstanding question of how lipid-enriched ascites reprogram MФs and provide a therapeutic target for reprogramming protumoral TAMs in EOC peritoneal metastases.

Recent studies have suggested that metabolic impacts such as hypoxia, nutrient availability, oncometabolite accumulation, etc., may induce metabolic disturbance and modulate the fitness of immune status within the ascitic microenvironmental niche [36, 43, 44]. Lipid enrichment is one of the significant metabolic hallmarks of ovarian cancer ascites [12]. It has been reported that unsaturated fatty acids, which are the primary constituents of lipid-rich ascites, are often associated with the adverse prognosis of patients with epithelial ovarian cancer (EOC) [12]. The elevation of unsaturated fatty acids has been shown to promote tumor metastasis via the recruitment of tumor-promoting immune cells, such as higher M2-like TAMs populations in the OCM/AS [45], suggesting an intimate association between the accelerated lipid metabolic activity and cancer immunity in modulating the tumor aggressiveness of metastatic ovarian cancer, which provided a novel therapeutic insight into cancer treatment via immunometabolic rewiring [46].

The bioactive lipid mediators derived PUFAs could modulate immune responses via various mechanisms, from cell membrane formation to signal transcription factors [47, 48]. Our study found that the dominant PUFAs, such as LA and MAA, have been shown to induce polarization of protumoral M2-like TAMs through modulating RhoA-YAP1 signaling. Our findings are in line with the previous studies reporting that conjugated linoleic acid (CLA), (conjugated linoleic acid is a collective term for a mixture of positional and geometric isomers of linoleic acid) [49], exhibits anti-inflammatory effects on atherosclerotic through accelerating M2-like MФs polarization [50]. Additionally, n-3 PUFAs such as linoleic acid (LA) and arachidonic acid (AA) have been shown to retard M1-like MФs polarization [48]. These reports, including ours, imply that PUFAs such as linoleic acid potentially facilitate a protumoral M2 phenotype instead of a tumoricidal M1-like phenotype, promoting tumor growth and progression.

RhoA-GTPases (RhoA) are crucial signaling proteins in triggering multiple immune functions [51]. Studies have shown that PUFAs inhibited the gene expression of HMG-CoA reductase (HMGCR), the critical rate-limiting enzyme of mevalonate signaling that positively regulated RhoA activity, thus that PUFAs downregulated RhoA through the suppression of HMGCR [52, 53]. The Rho-GTPase pathway has exhibited positive regulation of YAP1 transcriptional activities through GPCR-mediated extracellular signaling and the actomyosin pathway [54]. Likewise, our data demonstrated that LA, the most dominant PUFA, could mediate RhoA suppression and reduce YAP1 (both total and nuclear YAP1) in M2-like polarized MΦs in the AS or OCM. These findings support that PUFAs are crucial in suppressing HMGCR/RhoA, reducing YAP1 levels, and promoting M2-like TAM polarization.

Mammalian STE20-like 1/2 (MST1/2) and Large Tumor Suppressor 1/2 (LATS1/2) are the core components of the Hippo pathway. In the activated Hippo pathway, MST1/2 with Salvador 1 (SAV1) adaptor form in complex and activate the LATS1/2–MOB1 complexes, which in turn retain YAP/TAZ in the cytoplasm, suppressing their nuclear level and transcriptional activities [55]. Our findings showed that the F-actin level was downregulated, but there was no change in MST1 expression in TAMs co-cultured in PUFAs enriched medium. In fact, a similar finding has been reported [38, 56] suggesting that PUFAs suppressed RhoA activity, reducing YAP1 expression through the F-actin cytoskeleton. This Hippo-independent regulation in YAP1 activity parallels the conventional MST1/2-YAP1 signaling pathway. Hence, in this study, MST1/2 inactivation may counterbalance the RhoA-F-actin pathway’s downregulation of YAP1. On the other hand, based on our findings and the availability of druggable targets for the conventional Hippo pathway, we showed that the potent MST1/2 inhibitor XMU MP1 could reverse protumoral M2-like TAM polarization through the restoration of nuclear YAP1 in MΦs. Our data indicate that XMU-MP1 suppresses tumor metastasis in vivo, suggesting that YAP1 activation in macrophages may trigger a more potent antitumor immune response than its tumor-promoting effects. However, activating YAP1 in TME and other human ovarian cancer cells by XMU MP1 still needs further investigation. On the other hand, Amin Ardestani et al. have recently identified Neratinib, an FDA-approved metastatic HER2-positive breast cancer drug that is also a potent MST1 inhibitor with 98% MST1 inhibition. Neratinib has been shown to significantly block MST1 activation in β-cells and islets [57], implying that Neratinib has demonstrated potential as a clinically viable treatment for targeting ovarian cancer peritoneal metastases in the future.

The ‘feedback loop’ effect of YAP1 on MФs fate or function could be a key ‘controller’ that determines the prime antitumor immune response and infiltration in the PUFAs-enriched ascites microenvironment. A recent study reported that YAP1 is differentially expressed in M1 and M2-polarized MФs (i.e., the level of YAP1 is lower in M2-like but higher in M1-like polarized MФs) [58]. Our study corroborates this, showing that nuclear YAP1 depletion favors protumoral M2-like polarization, whereas its restoration shifts macrophages towards tumoricidal activity. In line with this, Yap1-deficient macrophages in cKO mice show increased susceptibility to ID8-derived tumor growth and metastasis, attributing to a higher M2-like/M1-like macrophage ratio and decreased CD8^+^ T cell infiltration in the ascites and omentum. This underscores the critical role of TAMs status in governing peritoneal metastases.

## Conclusions

Our study uncovers a new regulatory pathway in which a lipid-rich ascites microenvironment fosters protumoral M2-like TAM polarization through the PUFAs-triggered RhoA-YAP1 signaling cascade. Furthermore, we have shown that reinstating nuclear YAP1 by specifically inhibiting MST1/2 in TAMs can induce a shift in polarization from M2- to M1-like MФs polarization, offering a viable therapeutic strategy against epithelial ovarian cancer (EOC) peritoneal metastases (Supplementary Fig. S11).

### Electronic supplementary material

Below is the link to the electronic supplementary material.

Supplementary Material 2

## Data Availability

The RNA-seq data were deposited in the NCBI Gene Expression Omnibus database and can be accessed through accession number GSE175552. The data supporting the conclusions of this paper have been provided in this paper and the TCGA database. The raw data and the uncropped western blot images were deposited in Figshare DOI (10.6084/m9.figshare.22791209).

## Abbreviations

AS: Ascites fluid
ATP: Adenosine triphosphate
Arg1: Arginase 1
BSA: Bovine serum albumin
BMDMs: Bone marrow-derived macrophages
CD11b: Cluster of differentiation molecule 11B, also known as Integrin alpha M (ITGAM)
CD11c: Cluster of differentiation molecule 11 C, also known as Integrin, alpha X (ITGAX)
CCL2: Chemokine (C-C motif) ligand 2
CD14: Cluster of Differentiation 14
CD16: Cluster of Differentiation 16
CD19: Cluster of Differentiation 19
CD45: Cluster of Differentiation 45
CD56: Cluster of Differentiation 56
CD68: Cluster of Differentiation 68
CD86: Cluster of Differentiation 86
CD163: Cluster of Differentiation 163
CD206: Cluster of Differentiation 206
CM: Conditioned Medium
cKO: Conditional knockout
DCs: Dendritic cells
DMEM: Dulbecco’s Modified Eagle’s Medium
DMSO: Deoxyribonucleic acid
ECAR: Extracellular acidification rate
EOC: Epithelial ovarian cancer
FBS: Fetal Bovine Serum
FACS: Fluorescence-activated cell sorting
HMGCR: 3-Hydroxy-3-Methylglutaryl-CoA Reductase
LAST1/2: Large tumor suppressor kinase 1/2
IL4: Interleukin 4
IL10: Interleukin-10
IL13: Interleukin 13
ICB: Immune checkpoint blockade
IFN-γ: Interferon gamma
IHC: Immunohistochemical analysis
LA: Linoleic Acid
LPS: Lipopolysaccharides
LPMs: Large peritoneal macrophage
LPA: Lysophosphatidic acid
M0 MФs: Resting macrophages
MDSC: Myeloid-derived suppressor cells
MUFAs: Mono-Unsaturated fatty acids
MAA: Methyl arachidonate
MST1/2: Mammalian STE20-like protein kinase 1/2
m-IHC: Multiplexed Immunofluorescence
MFI: Mean fluorescence intensity
NOS2: Nitric Oxide Synthase 2
OXPHOS: Oxidative phosphorylation
OCM: Omental conditioned medium
OCM-C: Omental conditional medium pretreated with Cleanascites lipid remove reagent
OA: Oleic acid
OCR: Oxygen consumption rate
PFA: Paraformaldehyde Solution
PCA: Principal component analysis
PKA: Protein kinase A
PBS: Phosphate Buffered Saline
PUFAs: Poly-Unsaturated fatty acids
PVDF: Polyvinylidene difluoride
PBMC: Peritoneal blood mononuclear cells
PA: Palmitic acid
ROS: Reactive oxygen species
RNA: Ribonucleic Acid
RhoA: Ras Homolog Family Member A
Rot/Ant: Rotenone and antimycin
SDS: Sodium Dodecyl Sulphate
SFAs: Saturated fatty acids
SDS-PAGE: Sodium dodecyl sulfate polyacrylamide gel electrophoresis
SPMs: Small peritoneal macrophage
TAZ: Transcriptional co-activator with PDZ-binding motif
TME: Tumor microenvironment
TNF: Tumor necrosis factor
TAMs: Tumor associated macrophages
TCGA-OV: The Cancer Genome Atlas Ovarian Cancer
UFAs: Unsaturated fatty acids
WT: Wildtype
XMU: XMU MP1
YAP1: Yes-associated protein 1
