# Supplementary material for: Polyunsaturated fatty acids promote M2-like TAM deposition via dampening RhoA-YAP1 signaling in the ovarian cancer microenvironment
Source: Exp Hematol Oncol. 2024 Aug 28;13:90. doi: 10.1186/s40164-024-00558-8 (PMC11360340; doi:10.1186/s40164-024-00558-8)
Supplement: Supplementary file 1 — Supplementary Material 2 [file 40164_2024_558_MOESM1_ESM.docx]

**Supplementary Information for**

**Polyunsaturated Fatty Acids Promote M2-like TAM deposition via dampening RHOA-YAP1 signaling in the Ovarian Cancer Microenvironment**

Huogang WANG^1,5^, Mingo MH YUNG^1^, Yang XUAN^1^, Fushun CHEN^1^, Waisun CHAN^1^, Michelle KY SIU^1^, Runying LONG^1^, Shuo JIA^6^, Yonghao LIANG^2^, Dakang XU^4^, Zhangfa SONG^5^, Stephen KW Tsui^2^, Hextan YS NGAN^1^, Karen KL CHAN^1,*^ and David W CHAN^1, 2,3*^

^1^Department of Obstetrics & Gynaecology, LKS Faculty of Medicine, The University of Hong Kong, Hong Kong, China.

^2^School of Biomedical Sciences, The Chinese University of Hong Kong, Hong Kong, China.

^3^School of Medicine, The Chinese University of Hong Kong-Shenzhen, Shenzhen, China.

^4^Faculty of Medical Laboratory Science, Ruijin Hospital, School of Medicine, Shanghai Jiao Tong University, Shanghai 200030, China.

^5^Department of Colorectal Surgery, Sir Run Run Shaw Hospital, School of Medicine, Zhejiang University, Hangzhou, Zhejiang, 310016, P.R. China.

^6^Eye Center, The Second Affiliated Hospital, School of Medicine, Zhejiang University, 88 Jiefang Road, Hangzhou 310009, People’s Republic of China

*Correspondence should be addressed to:

Prof. David W CHAN, School of Medicine Start-up Building, CUHK-Shenzhen, 2001 Longxiang Blvd., Longgang District, Shenzhen 518172, China. Phone: (86) 755-23516153; E-mail: [dwchan@cuhk.edu.](mailto:dwchan@cuhk.edu.)cn, or Prof. Karen KL CHAN, Department of Obstetrics and Gynaecology, 6/F Professorial Block, Queen Mary Hospital, Pokfulam, Hong Kong, China. Phone: (852) 2255-4518; Fax: (852) 2255-0947; E-mail: kklchan@hku.hk


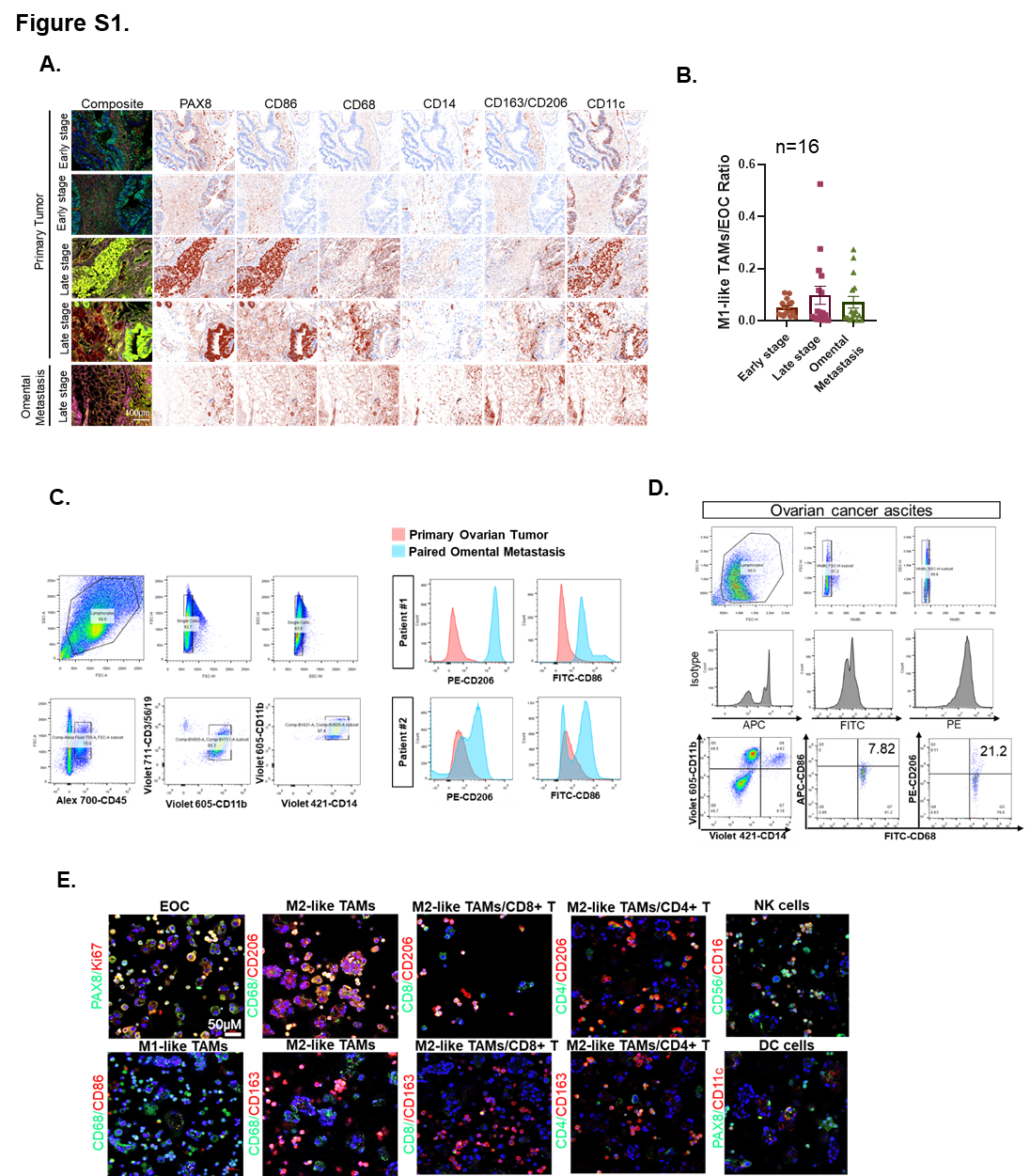


*Figure S1. The distribution of TAMs in EOC peritoneal metastases.*

**A.** Representative composite and single-stained IHC images of the m-IHC panel. Scale bar: 100μm. **B.** Ratio of M1-like TAMs to EOC. **C.** CD45^+^CD11b^+^CD14^+^CD3^-^CD56^-^CD19^-^ TAMs were isolated from the primary tumors and paired with omental metastatic tumors of patients with EOC. Comparison of the percentages of CD86^+^ and CD206^+^ TAMs derived from the primary and metastasis sites. N=2. **D.** Gating strategy for TAMs (CD14+CD11b^+^) derived from EOC ascites. **E.** Immunofluorescent staining of PAX8^+^Ki67^+^, CD68^+^CD86^+^, CD68^+^CD206^+^, CD68^+^CD163^+^, CD8^+^CD206^+^, CD8^+^CD163^+^, CD4^+^CD206^+^, CD4^+^CD163^+^, CD56^+^CD16^+^, PAX8^+^CD11c^+^ cells on malignant ascites spheroids from patients with EOC (n=3). Hoechst was used to stain nuclei. Scale bar: 50μm.


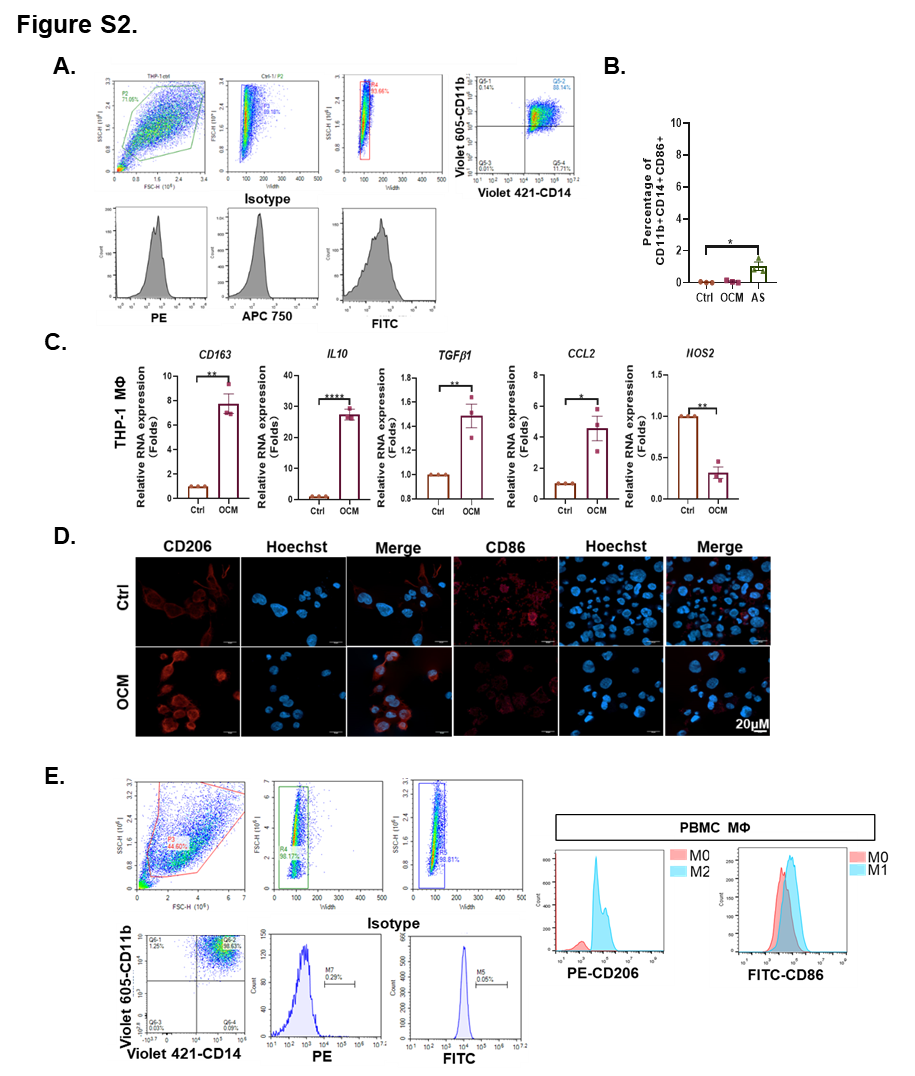


*Figure S2. MФs polarization in the ascites microenvironment.*

A. Gating strategy for CD14^+^CD11b^+^ MФs (THP-1 MΦs). B. The bar chart summarizes the expressions of CD11b^+^CD14^+^CD86^+^ MΦs (PBMC MФs) upon treatment with OCM or ascites (AS). C. QPCR analysis of *CD163, IL10, TGFβ, CCL2* and *NOS2* mRNA expression levels in M0 MΦs (THP-1) upon treatment with OCM or AS. The gene expression data were normalized to the reference gene *18S*, and are presented as the fold change relative to the control. D. Representative confocal microscopy images of CD86^+^ or CD206^+^ MΦs under Control (1% FBS RPMI) or OCM conditions. E. Gating strategy for flow cytometry analysis of CD14^+^CD11b^+^ MФs (PBMC MΦ) and the comparison of the percentages of CD206 and CD86 among M0 MΦs (PBMC MФs) stimulated with either control medium (1% FBS RPMI) or human LPS/IFNγ (20pg/mL, 20 ng/mL) or IL4/IL13 (20ng/mL, 20ng/mL).


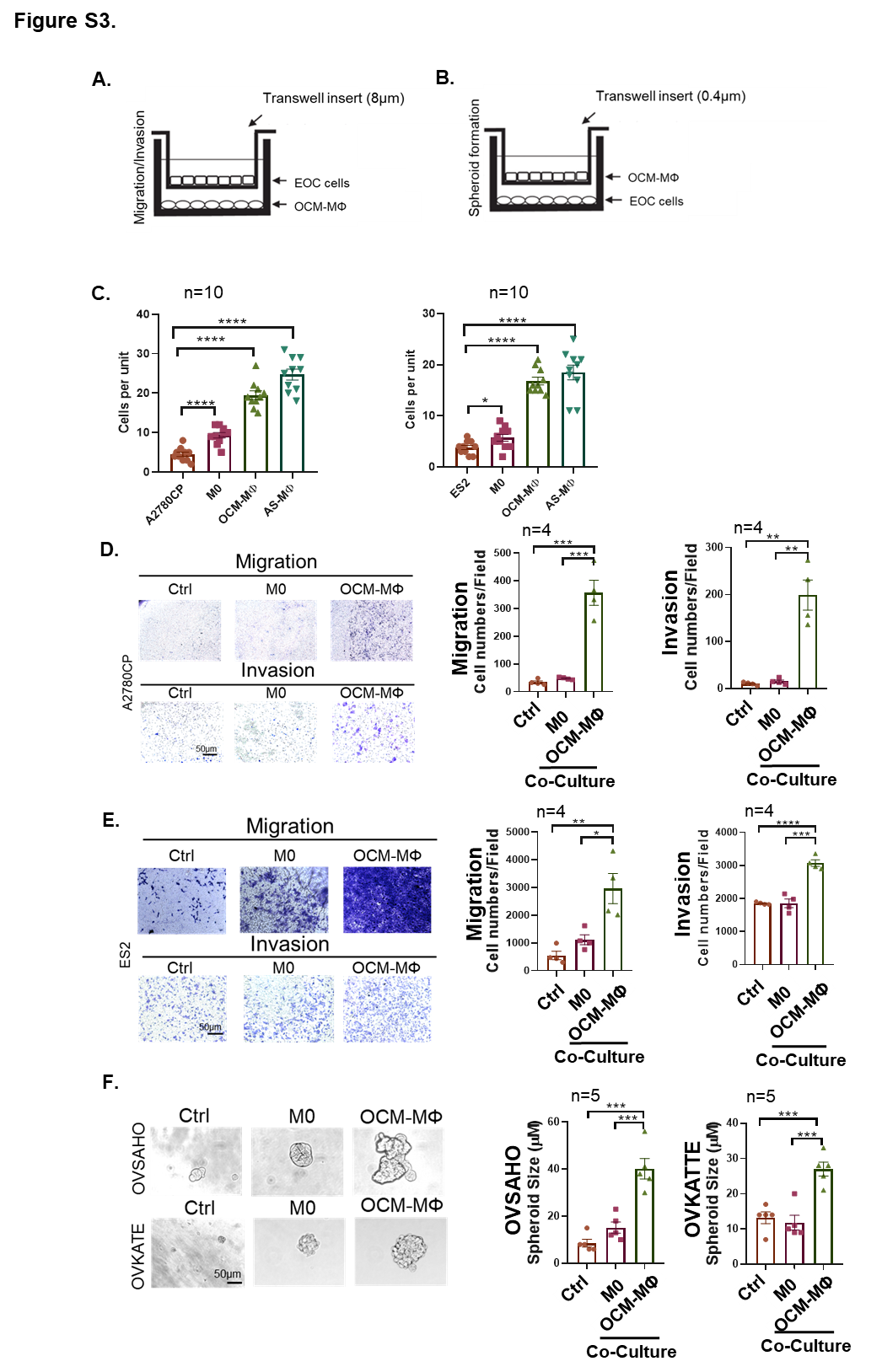


*Figure S3. MФs co-cultural model.*

**A.** Schematic representation of the migration/invasion experiment. **B.** Schematic representation of the spheroid formation experiment. **C.** Spheroid formation capacity of EOC cells alone or co-cultured with control MΦs or TAMs was determined by spheroid formation assays. The numbers of each spheroid were quantified as shown (n=10). D. EOC cells were seeded in the upper chamber, while M0 MΦs , OCM-MΦs, or no cells were seeded in the lower chamber of the transwell plates, and incubated for 36h. The stained cells were counted in four randomly selected fields. Representative images and quantitative results of cell migration and invasion were shown (n=4). Scale bar: 50μm. F. Spheroid formation capacity of EOC cells alone or co-cultured with control MΦs or TAMs was determined by spheroid formation assays. The size of the spheroids was quantified as shown (n=9). Scale bar: 50μm. The data were shown as the mean ± SEM and were analyzed by unpaired Student’s t-tests (n=5). The data are shown as the mean ± SEM and were analyzed by unpaired Student’s t-tests. The number of independent experiments was n=9, *, P ≤ 0.05; **, P ≤ 0.01; ***, P ≤ 0.001; ****, P ≤ 0.0001.


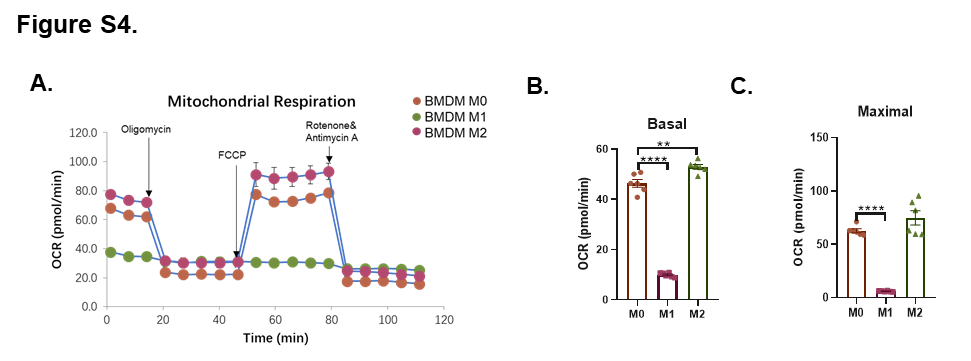


*Figure S4. The Metabolic patterns of M1-like and M2-like MФs.*

**A.** Representative measurements of the oxygen consumption rate (OCR) after the addition of oligomycin A (1 mM), FCCP (1.5mM), and rotenone and antimycin (Rot/Ant) (0.5mM) to resting MФs, M1-like MФs or M2-like MФs derived from murine BMDMs. **B.** Quantified basal OXPHOS. **C.** Maximal OXPHOS. The data are shown as the mean ± SEM and analyzed by unpaired Student’s t-tests. ***, P ≤ 0.001; ****; P ≤ 0.000


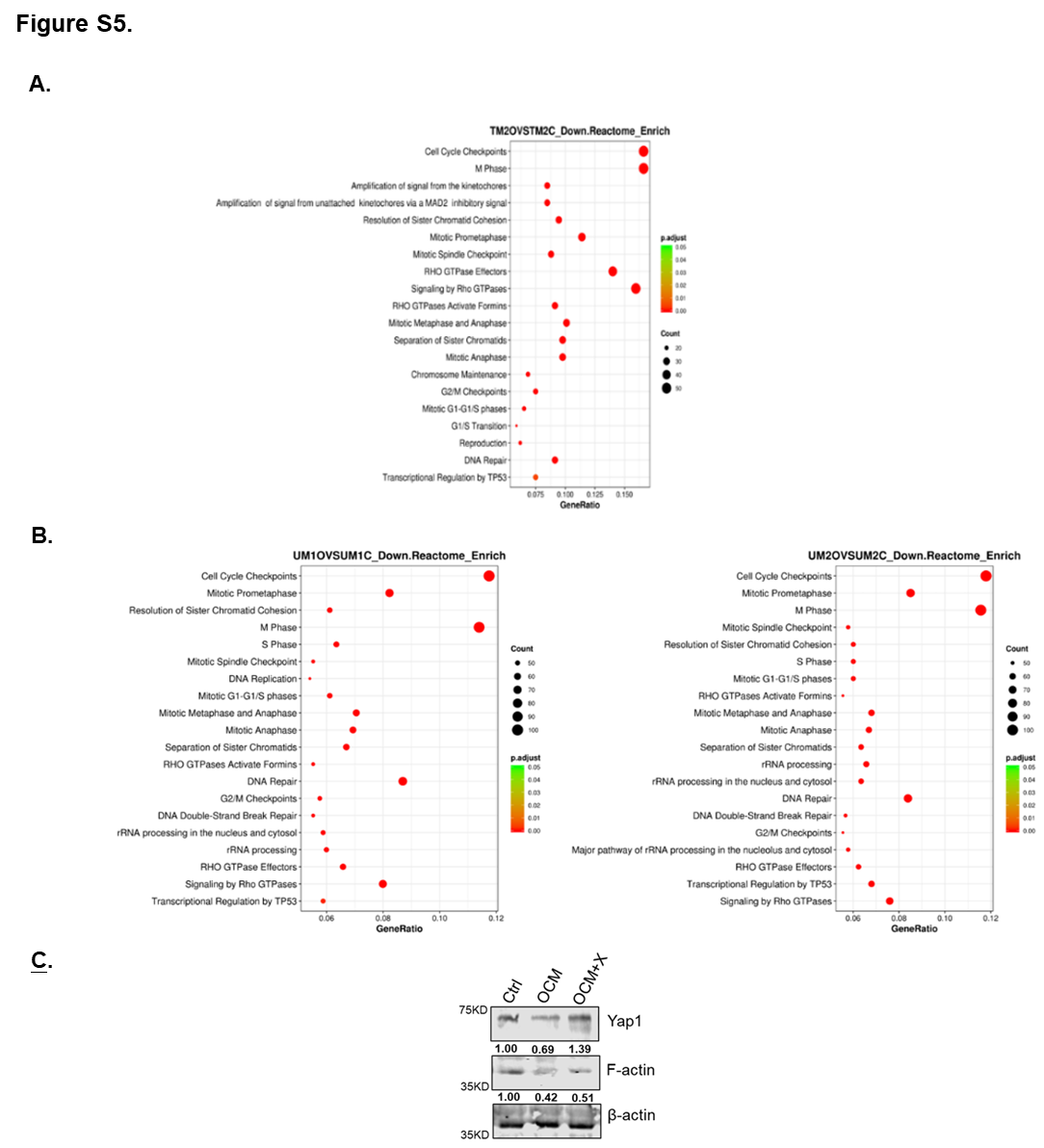


*Figure S5. PUFAs-enriched MAM downregulate the Rho-GTPase in polarized M1 or M2 MФs.* **A & B.** Top 20 significantly downregulated enriched pathways in the Reactome analysis results in M1 Фs with or without OCM coculture and M2 MФs with or without OCM coculture. M1 and M2 Фs were derived from THP-1 or U937 cells. C. OCM led to a reduction in YAP1 and F-actin expression in THP-1 MΦs after 24h treatment. XMU MP1 plus OCM treatment rescued partly YAP1 and F-actin expression in THP1 MΦs.

*
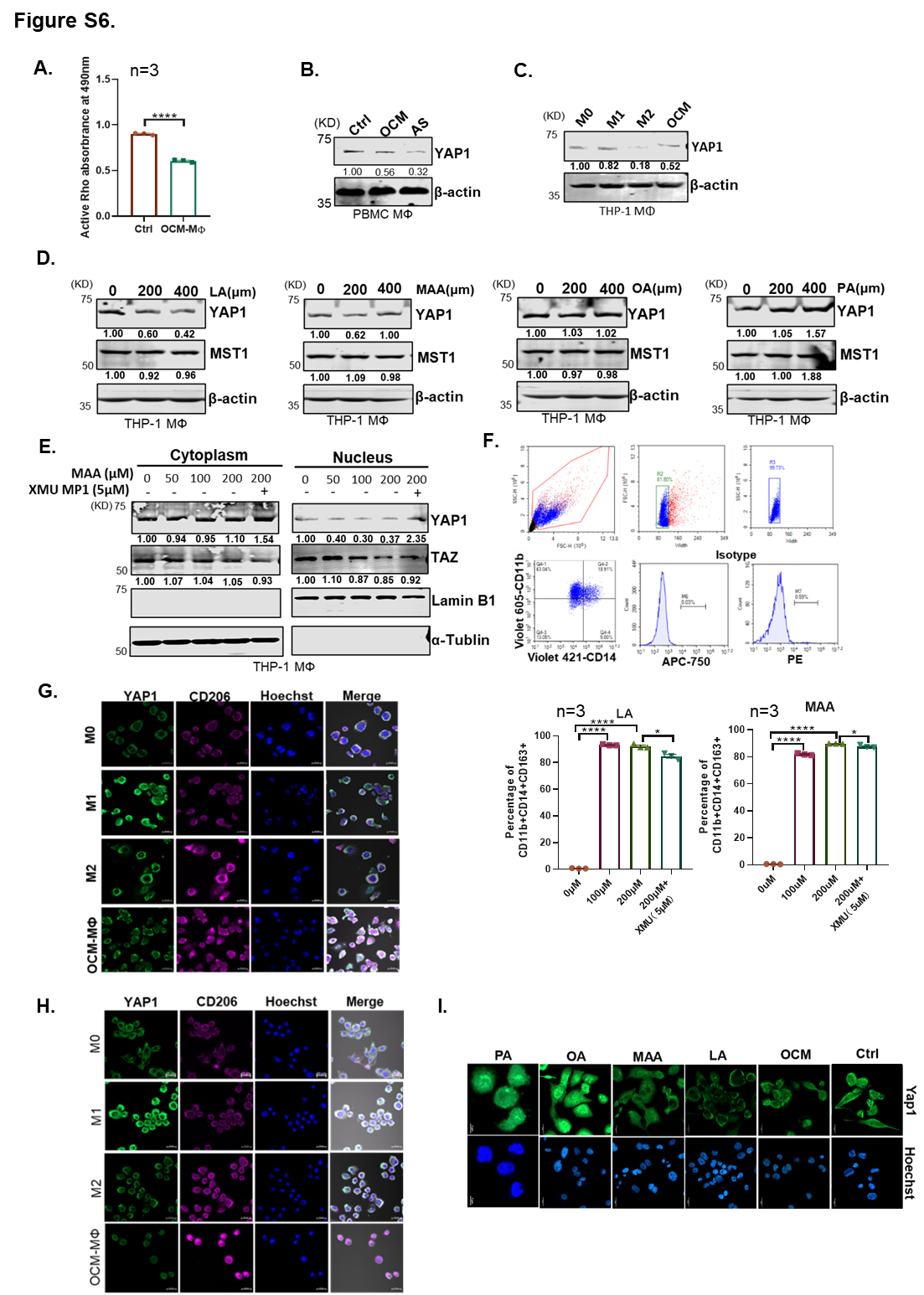
*

*Figure S6. PUFAs-enriched MAM modulates the RhoA-YAP1 axis in MΦs.*

**A.** G-LISA activation assays showed that active RhoA was attenuated in THP-1 MΦs co-cultured with OCM. **B.** OCM or AS reduced the expression level of YAP1 in M0 MФs (PBMC). **C.** M2 MФs or OCM MФs showed reduced YAP1 expression compared with M0 or M1 MФs. **D**. YAP1 expression was reduced by Linoleic acid (LA) only but not by methyl arachidonate (MAA), oleic acid (OA), or Palmitic acid (PA) in THP-1 MΦs. MST1 expression was not changed upon LA, MAA, OA and PA treatment. **E.** MAA reduced nuclear YAP1 activity in THP-1 MΦs after 24h treatment. XMU MP1 (5μM) restores nuclear YAP1 in MAA-treated MΦs. **F.** Gating strategy for CD14^+^CD11b^+^ MФs (THP-1 MΦ) and a bar chart summarizes the expression of CD11b^+^CD14^+^CD163^+^ cells among THP-1 MΦs after treatment with control (1%FBS/BSA RPMI), different doses of linoleic acid (LA), methyl arachidonate (MAA) or LA (200 μM), MAA(200 μM) in combined with XMU MP1 (5μM) for 48 h. The data are shown as the mean ± SEM and analyzed by unpaired Student’s t-tests.**,P ≤ 0.01; ***, P ≤ 0.001; ****; P ≤ 0.0001. **G.&H.** Immunofluorescence analysis YAP1 (green) in M0, M1, M2 and OCM MФs (THP-1 MΦs, U937 MΦs). Nuclei were counterstained with Hoechst (blue). Scale bar: 20μm. **I.** Immunofluorescence analysis YAP1 (green) in THP-1 MФs treated with OA, LA, MAA, PA, or control medium. Nuclei were counterstained with Hoechst (blue). Scale bar: 20μm.


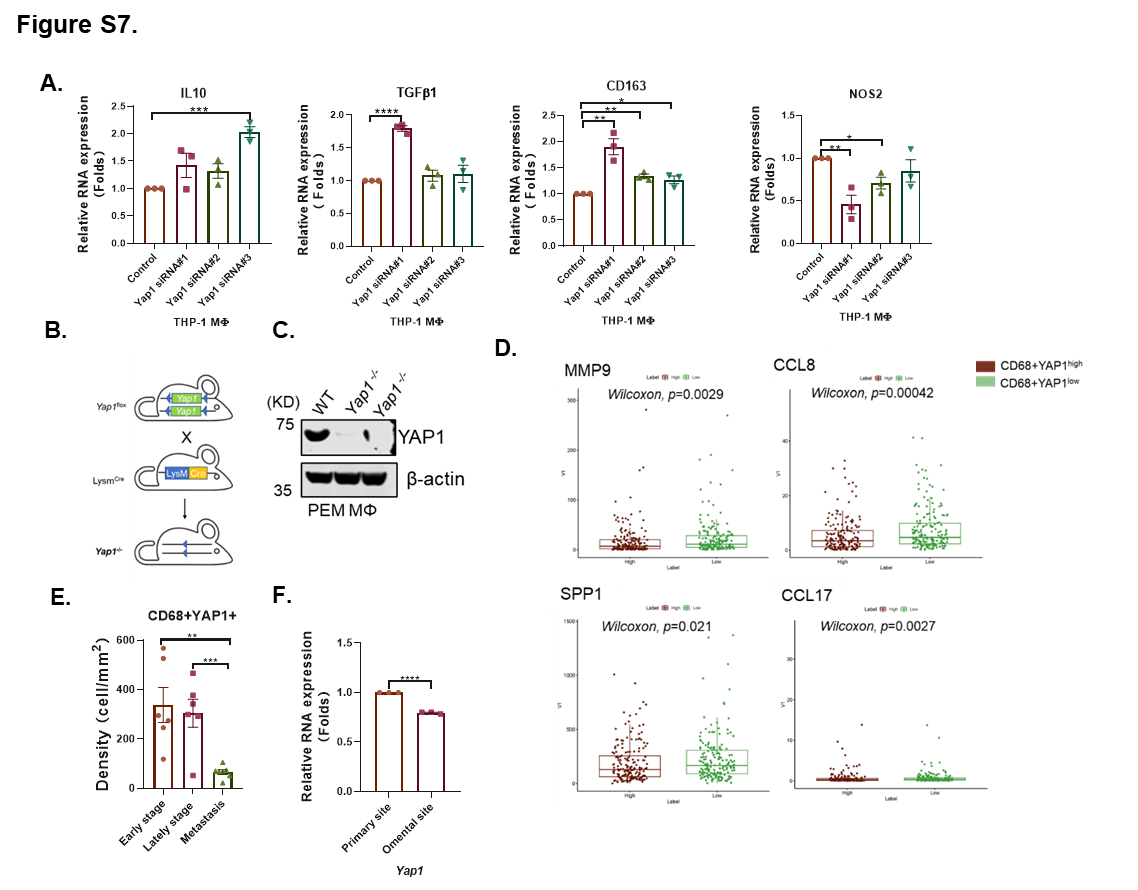


*Figure S7. Yap1-specific conditional knockout (cKO)in MФs determines TAM polarization in a mouse model.*

**A.** *CD163*, *IL10*, *TGFβ,* and *NOS2* mRNA expression levels were measured by qPCR in M0 MΦs treated with control siRNA or YAP1 siRNA. The gene expression data were normalized to the reference gene *18S*, and the results are presented as the fold change relative to the control. **B.** Schematic diagram shows the procedure of the generation of Yap1-cKO mice by crossbreeding Yap1^fl/fl^ and LysMcre mice. **C.** Yap1 expression at the protein level was assessed in peritoneal macrophages (PEMs) from WT and *Yap1^-/-^* mice. **D**. Expression distribution of the *MMP9*, *CCL8*, *SPP1* and *CCL17* gene in CD68^+^YAP1^high^ and CD68^+^YAP1^low^ ovarian cancers was investigated based on TCGA-OV cancer data. The significance of the two groups of samples passed the Wilcoxon test. **E.** Distinct distributions of CD68^+^YAP1^+^ TAMs population densities across regions in early stage (n=6), late stage (n=6), and omental metastasis (n=6). **F.** QPCR analysis of YAP1 mRNA expression levels in CD45^+^CD11b^+^CD14^+^CD3^-^CD56^-^CD19^-^TAMs were isolated from the primary tumor and paired with omental metastatic tumor of a patient with EOC. The gene expression data were normalized to the reference gene *18S* and are presented as the fold change relative to the control.


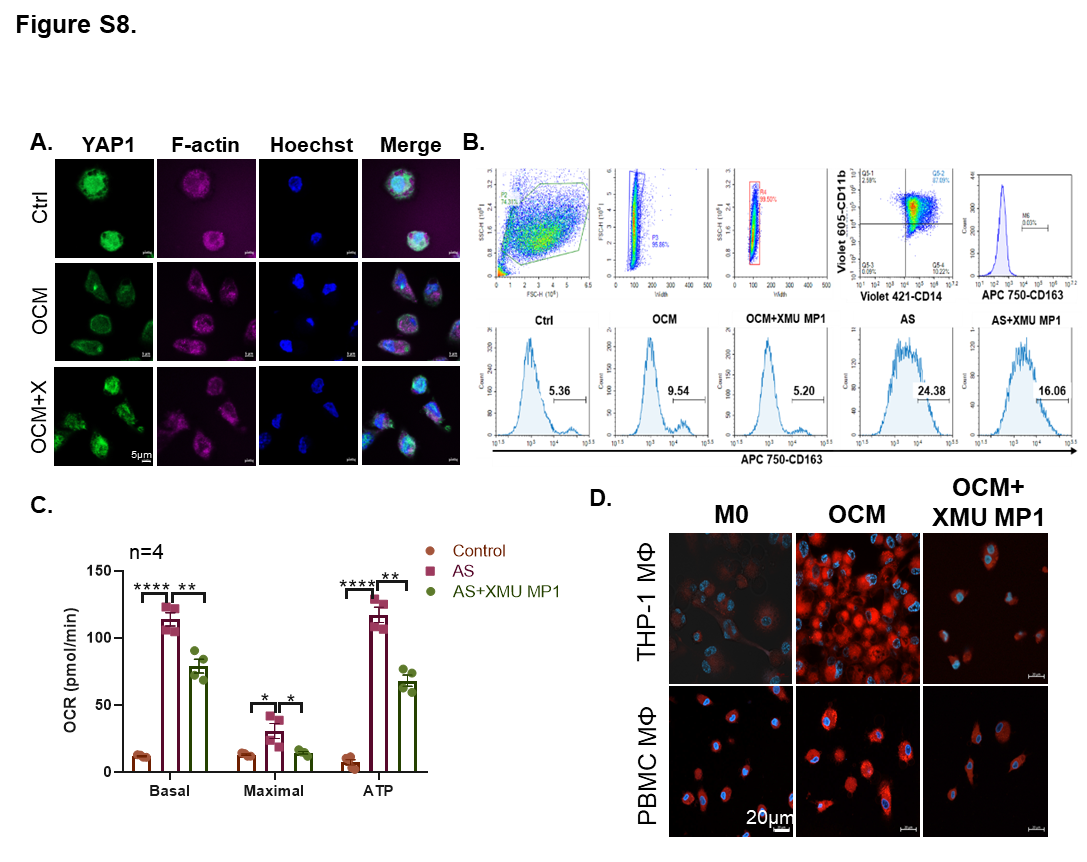


*Figure S8. XMU MP1 restores Yap1 expression in TAMs and promotes M1-like MФs elevation*

**A.** Immunofluorescence analysis YAP1 (green) in THP-1 MΦs embedded in OCM or OCM plus XMU MP1 (5μM). Nuclei were counterstained with Hoechst (blue). Scale bar: 5μm. **B.** Gating strategy for CD14^+^CD11b^+^ MФs (PBMC MΦ) and comparison of the percentages of CD163 among PBMC MΦs stimulated with control, OCM, OCM plus XMU MP1 (5μM), AS or AS plus XMU MP1 for 24h. **C.** Quantified basal OXPHOS and maximal and respiration-linked ATP in TAMs (PBMC MΦs treated with AS) with or without XMU MP1 (5μM) treatment. **D.** Lipid droplet formation assays revealed the formation of lipid droplets in MФs in control, OCM, or OCM plus XMU MP1 groups (5μM) groups. Scale bar: 20μM. The data are shown as the mean ±SEM and analyzed by unpaired Student’s t-tests.*, P ≤ 0.05; **,P ≤ 0.01; ***, P ≤ 0.001; ****; P ≤ 0.0001.


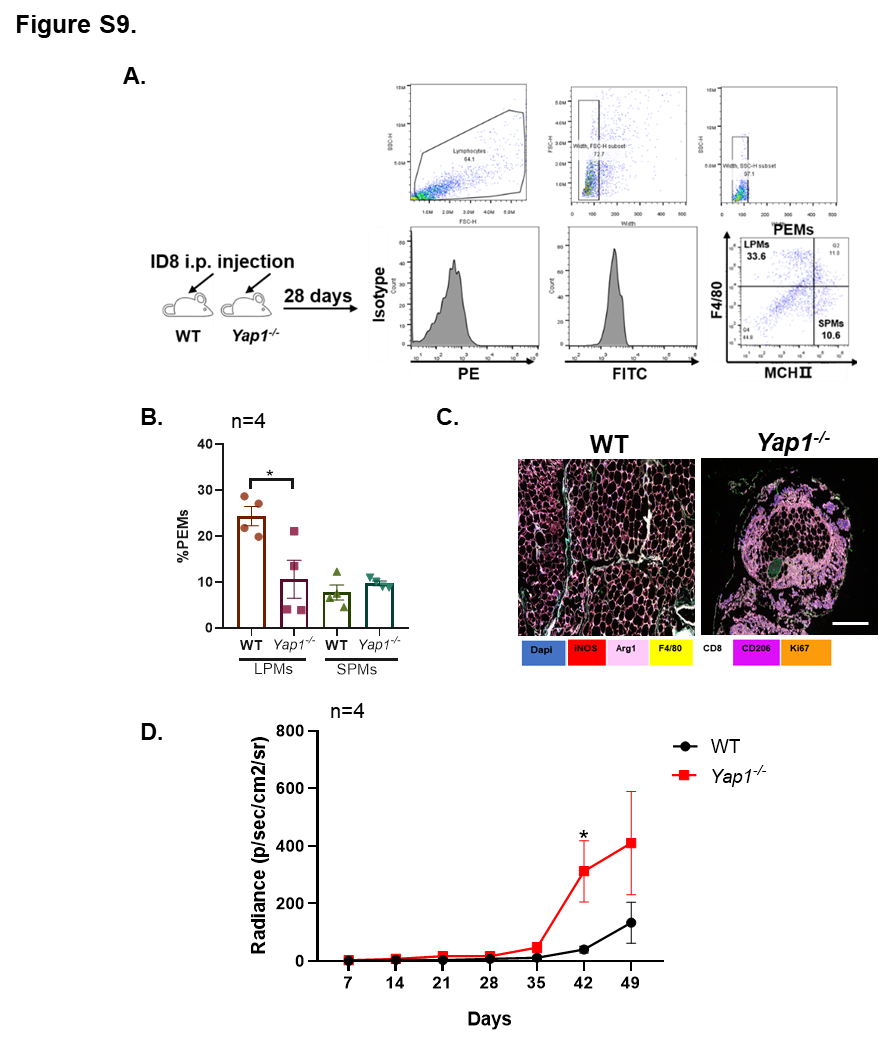


*Figure S9. MФs deficiency with Yap1 accelerates EOC peritoneal metastase progression.*

**A.** Gating strategy of murine PEMs by flow cytometry 28 days after intraperitoneal (*i.p.*) injection of ID8 cells. LPMs were subsequently gated as F4/80^+^ MHCII^-^, and SPMs were gated as F4/80^-^MHCII^+^. **B.** Percentages of LPMs and SPMs in WT and *Yap1^-/-^* mice. **C.** Representative composite images of the m-IHC panel in WT and *Yap1^-/-^* mice 28 days after intraperitoneal (*i.p.*) injection of ID8 cells. Scale bar: 100μm. **D.** ID8- GFP/Lucifer cells were intraperitoneally injected into WT and *Yap1^-/-^* mice. After seven days, the tumor burden was evaluated by bioluminescence imaging (n=4).

*
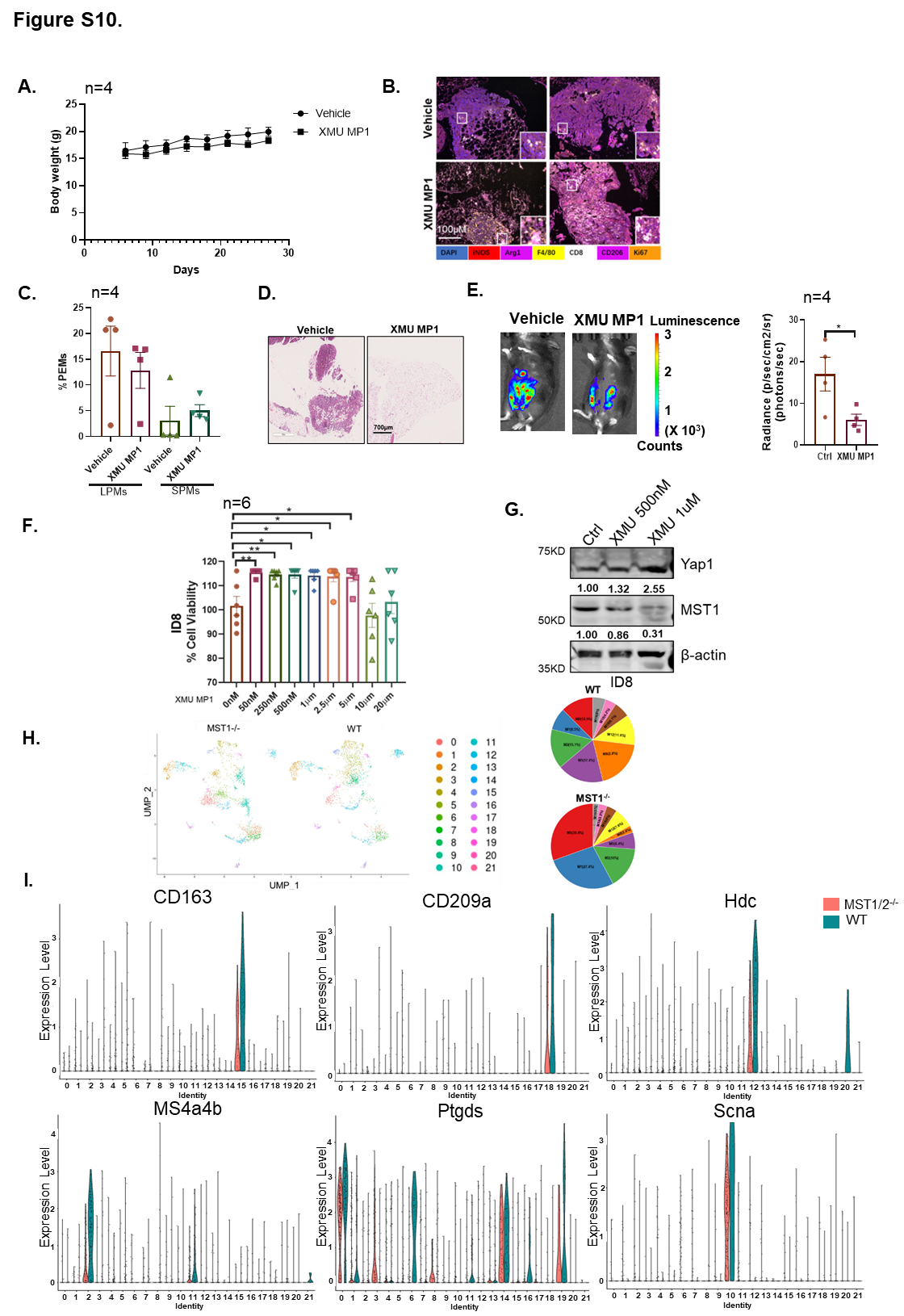
*

*Figure S10. XMU MP1 promotes M2-like TAMs reprogramming to M1-like MФs and inhibits EOC peritoneal metastases progression.*

**A.** Body-weight changes in mice that received XMU MP1 or vehicle for 28 days treatment. **B.** Representative composite images of the m-IHC panel in Vehicle and XMU MP1 treated mice. Scale bar: 100μm. **C.** Percentages of LPMs and SPMs in PEMs derived from vehicle- or XMU MP1-treated mice. The data are shown as the mean ± SEM and analyzed by unpaired Student’s t-tests.*, P ≤ 0.05. **D.** Representative H&E-stained images of omental metastases in tumor-bearing mice treated with vehicle- or XMU MP1 for 28 days. Scale bar: 700μm. **E.** Metastasis in Vehicle and XMU MP1 treated mice following *i.p.* injection was evaluated after 28 days by bioluminescence imaging. The results of fluorescence quantification are on the right. **F.** The murine ovarian cancer cell ID8 cells were incubated with either Vehicle or XMU MP1 in a dosed manner. Subsequently, the viability of ID8 cells was assessed using the XTT assay. The experiment was performed in triplicate (n=6). **G.** The protein expression of YAP1, MST1 were measured in ID8 cells treated with Vehicle or XMU MP1. The expression of β-actin as internal control. **H.** UMAP plot of immune cells from WT and Mst1/2^-/-^ mice (GSE181935), with each color-coded for 22 cell types and sample origin and Pie charts depicting the ratio of the seven decreased cell types in MST1/2^-/-^ mice. **I.** Violin plots show specifically expressed genes (SEGs) expression in decreased immune cell subtypes in MST1/2^-/-^ mice.


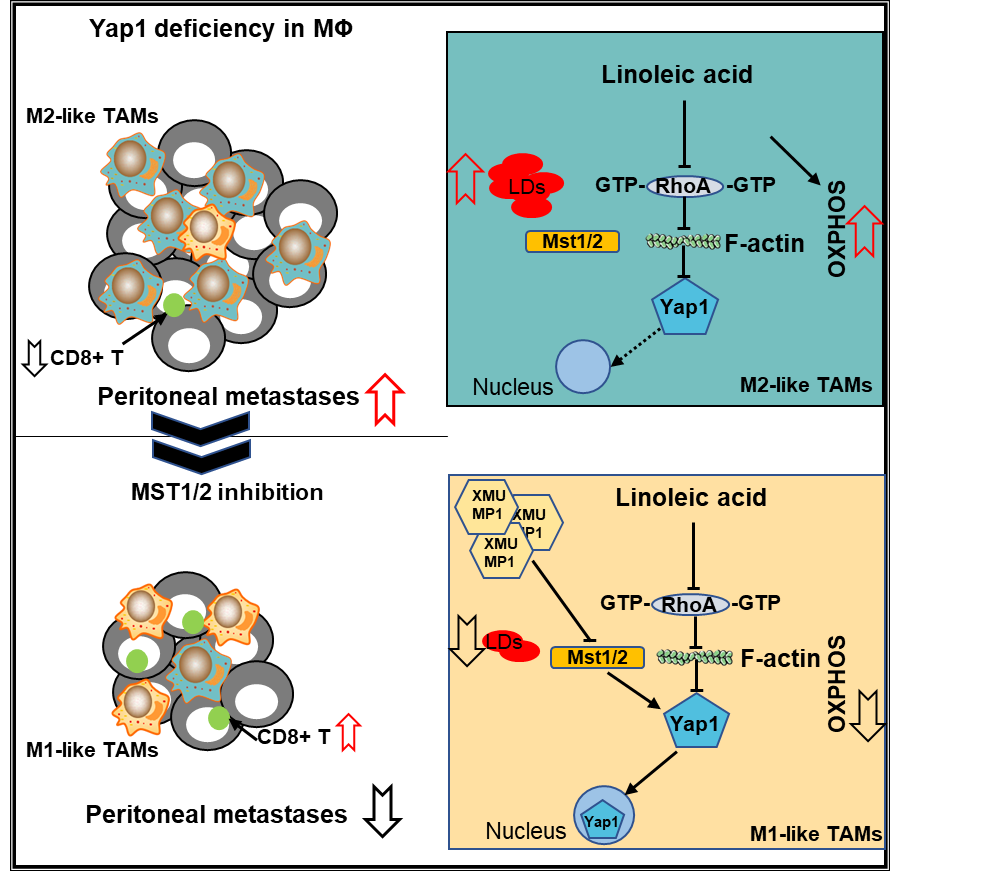


*Figure S11, Graph summary*

PUFAs-enriched OCM/ascites promote M2-like TAM deposition through inhibiting RhoA-YAP1 signaling, and M2-like TAMs with YAP1 deficiency accelerate peritoneal metastases of EOC with suppressive prometastatic microenvironment. XMU MP1 reprograms M2-like TAMs to M1-like polarized MФs through Yap1 activation, inhibits EOC peritoneal metastases, and elevates CD8+ T cell infiltration.

Table S1

| REAGENT or RESOURCE | SOURCE and Dilution | IDENTIFIER |
| --- | --- | --- |
| Antibodies | | |
| Rabbit anti-CD68 | Abcam; 1:300 | Cat#ab213363, RRID:AB_2801637 |
| Mouse anti-PAX8 | Santa Cruz Biotechnology  ;1:200 | Cat# sc-81353, RRID:AB_1127048 |
| Mouse anti-CD16 (2Q1240) | Santa Cruz Biotechnology  ;1:200 | Cat# sc-70548, RRID:AB_1120245 |
| Mouse anti-B7-2 (BU63) | Santa Cruz Biotechnology  ;1:200 | Cat# sc-19617, RRID:AB_627201 |
| Mouse anti-CD163 (GHI/61) | Santa Cruz Biotechnology  ;1:200 | Cat# sc-20066, RRID:AB_626933 |
| Mouse anti-CD206 (D1) | Santa Cruz Biotechnology  ;1:200 | Cat# sc-376108, RRID:AB_10987732 |
| Mouse anti-Integrin αX (B-6) | Santa Cruz Biotechnology  ;1:300 | Cat# sc-46676,  RRID:AB_626859 |
| CD56 | Santa Cruz Biotechnology  ;1:200 | Cat# sc-7326, RRID:AB_627127 |
| CD16 | Santa Cruz Biotechnology  ;1:200 | Cat# sc-20052, RRID:AB_626925 |
| Recombinant Anti-CD8 alpha antibody | Abcam ;1:200 | Cat# ab245118, RRID:AB_3068617 |
| F4/80 | Abcam ;1:300 | Cat# ab6640, RRID:AB_1140040. |
| Anti-CD8 alpha antibody | Abcam ;1:200 | Cat# ab209775, RRID:AB_2860566. |
| Ki67 | Abcam ;1:300 | Cat# ab16667, RRID:AB_302459. |
| iNOS | Abcam ;1:200 | Cat# ab178945, RRID:AB_2861417 |
| Arg1 | Abcam ;1:200 | Cat#: ab233548, RRID:AB_2895715 |
| CD163 | Abcam ;1:200 | Cat#: ab182422, RRID:AB_2753196 |
| CD206 | Abcam ;1:200 | Cat#: ab64693, RRID:AB_1523910 |
|  |  |  |
| Rabbit anti-YAP1 | Elabscience ;1:1000 | Cat# E-AB-62344,  RRID:AB_2893039 |
| Rabbit anti-Lamin B1 | Proteintech ;1:1000 | Cat# 12987-1-AP, RRID:AB_2136290 |
| Rabbit anti-MST1 | Cell Signaling Technology  ;1:1000 | Cat# 3682, RRID:AB_2144632 |
| Mouse anti- beta Actin | Thermo Fisher Scientific  ;1:5000 | Cat# MA5-15739, RRID:AB_10979409 |
| Mouse anti-Tubblin | Sigma; 1:5000 | Cat# T5168, RRID:AB_477579 |
| Anti-TAZ antibody | Abcam: 1:1000 | Cat# ab110239, |
| Rabbit anti-RhoA | Cell Signaling Technology  ;1:1000 | Cat #2117, RRID:AB_10693922. |
| Rabbit anti-Cdc42 | Cell Signaling Technology  ;1:1000 | Cat# 2466, RRID:AB_2078082 |
| Rabbit anti-RAC1 | Thermo Fisher Scientific  ;1:1000 | Cat# PA1-091, RRID:AB_2539856 |
| CD206-PE | BD Biosciences | Cat#555954, RRID:AB_396250 |
| CD86-PE | BD Biosciences | Cat# 562390, RRID:AB_11154047 |
| CD68-FITC | BF Pharmingen | Cat# 562117, RRID:AB_10896283 |
| CD86-FITC | eBioscience | Catalog # 11-0862-82, RRID:AB_465148 |
| CD45- Alexa Fluor 700 | Invitrogen™ | Cat # MHCD4529, RRID:AB_10373857 |
| CD3- Brilliant Violet 711 | Biolegend | Cat#300464, RRID:AB_2566036 |
| CD56- Brilliant Violet 711 | Biolegend | Cat#318336, RRID:AB_2562417 |
| CD19- Brilliant Violet 711 | Biolegend | Cat#302246, RRID:AB_2562065 |
| CD14-Brilliant Violet 421™ | Biolegend | Cat#301830, RRID:AB_10959324 |
| CD11b-Brilliant Violet 605™ | Biolegend | Cat#301332, RRID:AB_2562021 |
| CD163- APC/Fire™ 750 | Biolegend | Cat#333634, RRID:AB_2734333 |
| Mo-F4/80-APC | eBioscience | Cat# 14-4801-82, RRID:AB_467558 |
| Mo-CD86-FITC | eBioscience | Cat # 11-0862-82, RRID:AB_465148 |
| Mo-CD206-PE-eFluor 610 | eBioscience | Cat # 61-2061-82, RRID:AB_2802389 |
| MHCII- Alexa Fluor® 700 | Biolegend | Cat # 107622, |
| Biological samples |  |  |
| Buffy coat | Hong Kong Red Cross | NA |
| Ascites from ovarian cancer patients. | Queen Mary Hospital (Hong Kong) | HKU/HA HKW IRB) (Institutional Review Board number: UW 20-256) |
| Ovarian tumor tissues and omentum tissues | Queen Mary Hospital (Hong Kong) | HKU/HA HKW IRB) (Institutional Review Board number: UW UW11-298) |
| Chemicals, peptides, and recombinant proteins | | |
| Recombinant Human M-CSF | LPEPROTECH | Cat#300-25 |
| LPS | Enzo Life Sciences | Cat# 03271825 |
| Recombinant Human IFN-γ | PEPROTECH | Cat# 300-02 |
| Recombinant Human IL-13 | PEPROTECH | Cat# 200-13 |
| Recombinant Human IL-4 | PEPROTECH | Cat# 200-04 |
| Phorbol 12-myristate 13-acetate | Abcam | Cat# ab120297 |
| Liberase enzyme TL | Roche | Cat#5401020001 |
| Liberase enzyme DL | Roche | Cat#5401160001 |
| Dnase 1 | Sigma Aldrich | Cat#11284932001 |
| XMU-MP-1 | MOLNOVA | Cat# M13204 |
| Oleic acid | Sigma Aldrich | Cat# O1008 |
| Methyl arachidonate | Sigma Aldrich | Cat# A9298 |
| Palmitic acid | Sigma Aldrich | Cat# P0500 |
| Linoleic acid | Sigma Aldrich | Cat# L1376 |
| Recombinant mouse M-CSF | PEPROTECH | Cat# 315-02 |
| Recombinant mouse IFN-γ | PEPROTECH | Cat# 315-05 |
| Lipopolysaccharides from Escherichia coli O55:B5, | Sigma Aldrich | Cat# L2880-25MG |
| Recombinant mouse IL13 | PEPROTECH | Cat# 210-13 |
| Recombinant mouse IL4 | PEPROTECH | Cat# 214-14 |
| Critical commercial assays | | |
| Cellular ROS Assay Kit | Abcam | Cat# ab113851 |
| Opal 7-Color IHC Kits | AKOYA BIOSCIENCES | Cat# NEL811001KT |
| Opal-480 dye | AKOYA BIOSCIENCES | Cat# FP1500001KT |
| Macrophage Isolation Kit (Peritoneum) | Miltenyi Biotec | Cat# 130-110-434 |
| human CD14 Microbeads | Miltenyi Biotec | Cat# 130-050-201 |
| Density gradient medium for the isolation of mononuclear cells | Lymphoprep^TM^ | Cat# 07851 |
| ImmunoCult^TM^-SF Macrophage Medium | STEMCELL | Cat# 10961 |
| G-LISA RhoA Activation Assay | Cytoskeleton, Inc. | Cat# BK124-S |
| Active Rho Pull-Down and Detection Kit | ThermoFisher Scientific | Cat# 16116 |
| Seahorse XF Cell Mito Stress Test Kit | Agilent Technologies | Cat# 103015-100 |
| NE-PER™ Nuclear and Cytoplasmic Extraction Reagents | Thermo Fisher Scientific | Cat# 78833 |
| FuGene HD Transfection Reagent | Promega | Cat# E2311 |
| Cell Proliferation Kit II (XTT) | Roche | Cat# 11465015001 |
| Deposited data | | |
| RNA-seq data | This paper | GSE175552 |
| scRNA data | Public | GSE181935 |
| TCGA-OV |  |  |
| Experimental models: cell lines | | |
| THP-1 | ATCC | ATCC TIB-202 |
| U937 | ATCC |  |
| ID8 | Dr. Katherine F. Roby |  |
| OVKATE | JCRB |  |
| A2780CP | Prof. Benjamin Tsang |  |
| OVSAHO | JCRB |  |
| ES2 | ATCC |  |
| Experimental models: organisms/strains | | |
| Mouse: C57BL/6J |  |  |
| Mouse: YAP fl/fl |  |  |
| Mouse: Lysm^cre/cre^ |  |  |
| Oligonucleotides | | |
| qRT-PCR taqman probes, see Table S2 | ThermoFisher Scientific | Table S1 |
| YAP1 Human siRNA Oligo Duplex | ORIGENE | Cat# SR323110 |
| RHOA Human siRNA Oligo Duplex | Origene | Cat# TF309823 |
| Software and algorithms | | |
| Prism | Graphpad | https://www.graphpad.com/ |
| FlowJo | Flowjo | https://www.flowjo.com/ |
| Phenochart 1.0.9 | PerkinElmer | https://www.perkinelmer.com.cn |
| Image Studio Lite Ver 5.2 | LI-COR | https://www.licor.com |
| InForm 2.4.2 | PerkinElmer | https://www.perkinelmer.com.cn/ |
| Image J |  | https://imagej.nih.gov/ij/ |
| Other | | |
| Cleanascite™ Lipid Removal Reagent | Biotech Support Group, Monmouth  Junction, NJ, USA | Cat# X2555-50 |
| ACK Lysing Buffer | Gibco | Cat# 2187329 |

Taqman primer related to this Manuscript. Table S2

| Gene Symbol | Assay ID |
| --- | --- |
| CD163 | Hs00174705_m1 |
| IL10 | Hs00961622_m1 |
| NOS2 | Hs01075529_m1 |
| TGFβ1 | Hs00998133_m1 |
| CCL2 | Hs00234140_m1 |
| TNF | Hs00174128_m1 |
| Mst1 | Hs00360684_m1 |
| Yap1 | Hs05045510_s1 |
| Arg1 | Mm00475988_m1 |
| Fizz1 | Mm00445109_m1 |
| Ym1 | Mm00657889_mH |
| 18s | Hs03003631_g1 |
| GAPDH | Mm99999915_g1 |
